# Digital Health Interventions to Prevent Type 2 Diabetes Mellitus: Systematic Review

**DOI:** 10.2196/67507

**Published:** 2025-04-25

**Authors:** Tuan Duong, Quita Olsen, Anish Menon, Leanna Woods, Wenyong Wang, Marlien Varnfield, Lee Jiang, Clair Sullivan

**Affiliations:** 1 Queensland Digital Health Centre Faculty of Medicine The University of Queensland Brisbane Australia; 2 Family Medicine Department Hue University of Medicine and Pharmacy Hue University Hue Vietnam; 3 Princess Alexandra Hospital Metro South Hospital and Health Service Brisbane Australia; 4 Logan Hospital Metro South Hospital and Health Service Brisbane Australia; 5 Australian e-Health Research Centre Commonwealth Scientific and Industrial Research Organisation Brisbane Australia; 6 School of Public Health The University of Queensland Brisbane Australia; 7 Metro North Hospital and Health Service Brisbane Australia

**Keywords:** digital health, type 2 diabetes, prediabetes, prevent, digital health intervention, PRISMA

## Abstract

**Background:**

Digital health interventions (DHIs) have rapidly evolved and significantly revolutionized the health care system. The quadruple aims of health care (improving population health, enhancing consumer experience, enhancing health care provider [HCP] experience, and decreasing health costs) serve as a strategic guiding framework for DHIs. It is unknown how DHIs can impact the burden of type 2 diabetes mellitus (T2DM), as measured by the quadruple aims.

**Objective:**

This study aimed to systematically review the effects of DHIs on improving the burden of T2DM, as measured by the quadruple aims.

**Methods:**

PubMed, Embase, CINAHL, and Web of Science were searched for studies published from January 2014 to March 2024. Primary outcomes were the development of T2DM, hemoglobin A_1c_ (HbA_1c_) change, and blood glucose change (dysglycemia changes). Secondary outcomes were consumer experience, HCP experience, and health care costs. Outcomes were mapped to the quadruple aims. DHIs were categorized using the World Health Organization’s DHI classification. For each study, DHI categories were assessed for their effects on each outcome, categorizing the effects as positive, negative, or neutral. The overall effects of each DHI category were determined by synthesizing all reported positive, neutral, or negative effects regardless of the number of studies supporting each effect. The Cochrane risk-of-bias version 2 (RoB 2) tool for randomized trials was used to assess the quality of randomized controlled trials (RCTs), while the ROBINS-I (risk of bias in nonrandomized studies of interventions) tool was applied for nonrandomized studies.

**Results:**

In total, 53 papers were included. For the T2DM development outcome, the effects of DHIs were positive in 1 (1.9%) study and neutral in 9 (17%) studies, and there were insufficient data to assess in 4 (7.5%) studies. For the dysglycemia outcome, the effects were positive in 23 (43.4%) studies and neutral in 24 (45.3%) studies, and there were insufficient data in 6 (11.3%) studies. There were mixed effects on consumer experience (n=13, 24.5%) and a lack of studies reporting HCP experience (n=1, 1.9%) and health care costs (n=3, 5.7%). All studies that reported positive population health outcomes used a minimum of 2 distinct categories of DHIs. Among these successful studies, the one that reported delaying the development of T2DM and 16 (69.6%) of those reporting improvements in dysglycemia involved HCP interaction. Targeted communication with persons (TCP), personal health tracking (PHT), and telemedicine (TM) showed some evidence as a potentially useful tool for T2DM prevention and dysglycemia.

**Conclusions:**

The effects of DHIs on T2DM prevention, as measured by the quadruple aims, have not been comprehensively assessed, with proven benefits for population health, mixed results for consumer experience, and insufficient studies on HCP experience and health care costs. To maximize their effectiveness in preventing T2DM and managing dysglycemia, DHIs should be used in combination and strategically integrated with in-person or remote HCP interaction.

**Trial Registration:**

PROSPERO CRD42024512690; https://www.crd.york.ac.uk/PROSPERO/view/CRD42024512690

## Introduction

Type 2 diabetes mellitus (T2DM) is a growing health problem worldwide that affects all income levels and puts a heavy burden on health care systems [1]. The increased prevalence of T2DM is largely due to changes in diet, rising obesity rates, and decreased physical activity [2]. Therefore, improving lifestyle can potentially help prevent T2DM [1]. Diabetes prevention programs (DPPs) and other lifestyle modifications strategies have been applied worldwide, demonstrating that such changes can effectively reduce the risk of developing T2DM [2-6].

Digital health interventions (DHIs), the application of digital technologies in health care [7], have transformed how health care is provided and experienced, leading to great health system efficiencies and clinical benefits [7-9]. Given the diverse communities involved in DHI (ie, technologists, researchers, clinicians, consumers, and government stakeholders), there is a need to establish a common language among these groups [7]. To address this need, the World Health Organization (WHO) developed a DHI classification system to provide a shared framework for naming, grouping, and evaluating DHIs. According to this classification, each DHI is categorized into groups based on primary users: persons, health care providers (HCPs), health system managers, and data services [7].

DHIs have been extensively applied in chronic disease management, showing clinical outcome improvements, better management, and cost savings [10-15]. During the past 10 years, DHIs are being increasingly applied in T2DM prevention, such as text messaging, web-based systems, telemedicine (TM), mobile health, software, wearables, and artificial intelligence (AI) [16,17]. A systematic review by Van Rhoon et al [18] in 2020 demonstrated that DHIs significantly reduce weight, enhance dysglycemia, and decrease T2DM incidence. According to the systematic review by Nguyen et al [19] in 2024, DHIs show further enhanced efficacy in preventing T2DM, highlighting the success of computer-based and mobile health in weight reduction, hemoglobin A_1c_ (HbA_1c_) improvement, and T2DM incidence reduction.

The quadruple aims of health care are the overarching goals focusing on improving population health, enhancing consumer experience, improving HCP experience, and decreasing health costs [20]. The quadruple aims have been regarded as a strategic compass in guiding DHIs in different contexts, such as chronic disease prevention [21], diagnosis and treatment [22,23], health care delivery [24], planning or decision-making [25], and managing unique health care challenges, such as the COVID-19 pandemic [26].

The impacts of DHIs on improving the burden of T2DM, as measured by the quadruple aims, still remain largely unknown. Our aim was to systematically review the current literature to examine the effects of DHIs on reducing the burden of T2DM, as measured by the quadruple aims.

## Methods

### Study Design

This systematic review was conducted and reported following PRISMA (Preferred Reporting Items for Systematic Reviews and Meta-Analyses) guidelines of 2020 [27]. Details of the PRISMA 2020 checklist are shown in Multimedia Appendix 1 [27]. The protocol of our systematic review was prospectively registered at PROSPERO (International Prospective Register of Systematic Reviews; registration number CRD42024512690)*.* Minor changes were made to the registration information: the title was updated, the quadruple aims were added to the objectives, and WHO’s DHI classification was included in the data synthesis.

### Ethical Considerations

Our study was a systematic review that used nonidentifiable, secondary data from published studies. According to institutional policies, no ethics review was required.

### Search Strategy and Selection Criteria

The PubMed, Embase, CINAHL, and Web of Science databases were searched with keywords in both Medical Subject Headings and title/abstract formats: “digital health interventions,” “type 2 diabetes,” and “prevention.” The review included only studies conducted in the past 10 years, from January 2014 to March 2024 (due to the emerging nature of the digital transformation in health care). Building, testing, and finalizing the search approach were performed by the research team, in consultation with 2 research librarians from the University of Queensland (Multimedia Appendix 2).

Inclusion and exclusion criteria are listed in Table 1. The intervention of interest was defined as the use of DHIs in support of the prevention of T2DM in individuals with monitored blood glucose or HbA_1c_. Given our focus on assessing the effects of DHIs on preventing or delaying the onset of T2DM, and considering that the diagnostic criteria for T2DM include HbA_1c_, blood glucose, and clinical criteria, our primary outcomes were the development of T2DM, HbA_1c_ change, or blood glucose change (dysglycemia changes). Secondary outcomes were consumer experience, HCP experience, and health care costs. Quantitative and qualitative data were included in our review. Studies were excluded if individuals or populations had known diabetes (T2DM, type 1 diabetes, or gestational diabetes, as described by authors of specific studies) or outcomes that did not report the development of T2DM, HbA_1c_ change, or blood glucose change.

**Table 1:** Systematic review inclusion and exclusion criteria.

Factor	Inclusion criteria	Exclusion criteria
Population	Individuals and populations who had blood glucose or HbA_1c_^a^ monitored	People with known diabetes (T2DM^b^, type 1 diabetes, or gestational diabetes)
Intervention	The use of DHI^c^ in support of prevention of T2DM	Not meeting inclusion criteria
Study design	RCTs^d^, non-RCTs, historically controlled studies, before-after studies, observational studies (cohort, case-control, cross-sectional studies), conference papers	Review studies, incomplete studies, full text not available
Comparator	Different DHI methods, routine care, or no comparator	No exclusions
Outcome	Primary outcomes: Development of T2DM HbA_1c_ change Blood glucose change Secondary outcomes: Consumer experience: any qualitative or quantitative measure of all interactions, influenced by an organization’s culture, that shape consumer perceptions across the DHI [28]; experience with the HCP^e^, consumer satisfaction, and experience with the entire DHI system [29] HCP experience: any qualitative or quantitative measure of all interactions and perceptions of HCPs regarding DHIs, such as the work environment, organizational culture, colleagues, and consumers [30] Health care costs: costs for consumers, organizations, or society, directly or indirectly, due to the implementation of DHIs [31]	Not reporting the development of T2DM, HbA_1c_ change, or blood glucose change
Publication year	2014-2024	N/A^f^
Language	English	Other languages

^a^HbA_1c_: hemoglobin A_1c_.

^b^T2DM: type 2 diabetes mellitus.

^c^DHI: digital health intervention.

^d^RCT: randomized controlled trial.

^e^HCP: health care provider.

^f^N/A: not applicable.

### Study Selection Process

Five reviewers participated in the selection and data extraction processes (authors TD, WW, QO, and LW as primary reviewers and author CS as a senior reviewer). All papers retrieved from the database were collected and imported to EndNote version 20 (Clarivate) before being uploaded to Covidence version 2. Titles and abstracts of identified studies were screened twice (by TD, WW, QO, and LW) for potential eligibility using the inclusion and exclusion criteria. Full texts that met the inclusion criteria were retrieved and independently evaluated for their eligibility by the reviewers. Duplicates identified either automatically by Covidence or manually were excluded. Reasons for excluding full-text papers were reported. Any disagreements were solved via discussion and consensus.

A data extraction form was developed by the research team and uploaded to Covidence (Multimedia Appendix 3). Data from the selected papers were extracted and then checked (TD and QO). Any discordance was resolved via discussion and consensus of the reviewers.

### Statistical Analysis

Primary and secondary outcomes were mapped to the quadruple aims, which included improving population health, enhancing consumer experience, enhancing HCP experience, and reducing health costs. The population health aspect was measured by the development of T2DM and dysglycemia changes (changes in HbA_1c_ or blood glucose).

Using WHO’s DHI classification (2023 version) [7], DHIs were classified into different categories, such as targeted communication with persons (TCP) for targeted individuals, untargeted communication with persons for undefined groups, person-to-person communication (PPC) in networks or forums, personal health tracking (PHT) for self-monitoring, on-demand communication with persons (DCP) for accessing health information, person-centered health records (PHR), HCP decision support (DS), and TM [7]. Details of the classification are shown in Multimedia Appendix 4.

For each study, DHI categories were assessed for their effects on each outcome, categorizing the effects as positive, negative, or neutral:

- Quantitative data: Effects were reported as “positive” if there was a statistically significant improvement in outcomes, “negative” if outcomes statistically worsened, “neutral” if there was no statistically significant impact, and “not available” if data were insufficient for evaluation.
- Qualitative data (consumer experience and HCP experience outcomes): Effects were reported as “positive” if there was only positive feedback, “negative” if there was only negative feedback, “mixed” if there was both positive and negative feedback, “neutral” if there was no positive and negative feedback, and “not available” if data were insufficient for evaluation.

The overall effects of each DHI category were determined by synthesizing all reported positive, neutral, or negative effects regardless of the number of studies supporting each effect.

A meta-analysis was not performed because of the numerous heterogeneous study designs with different interventions or outcomes.

### Risk-of-Bias Assessment

The Cochrane risk-of-bias version 2 tool (RoB 2) for randomized trials was used to assess the quality of RCTs [32], while the ROBINS-I (risk of bias in nonrandomized studies of interventions) tool was applied for nonrandomized studies [33] by TD, QO, and LJ. Any disagreements were solved via discussion and consensus.

## Results

### Characteristics of Included Studies

**Figure 1:**
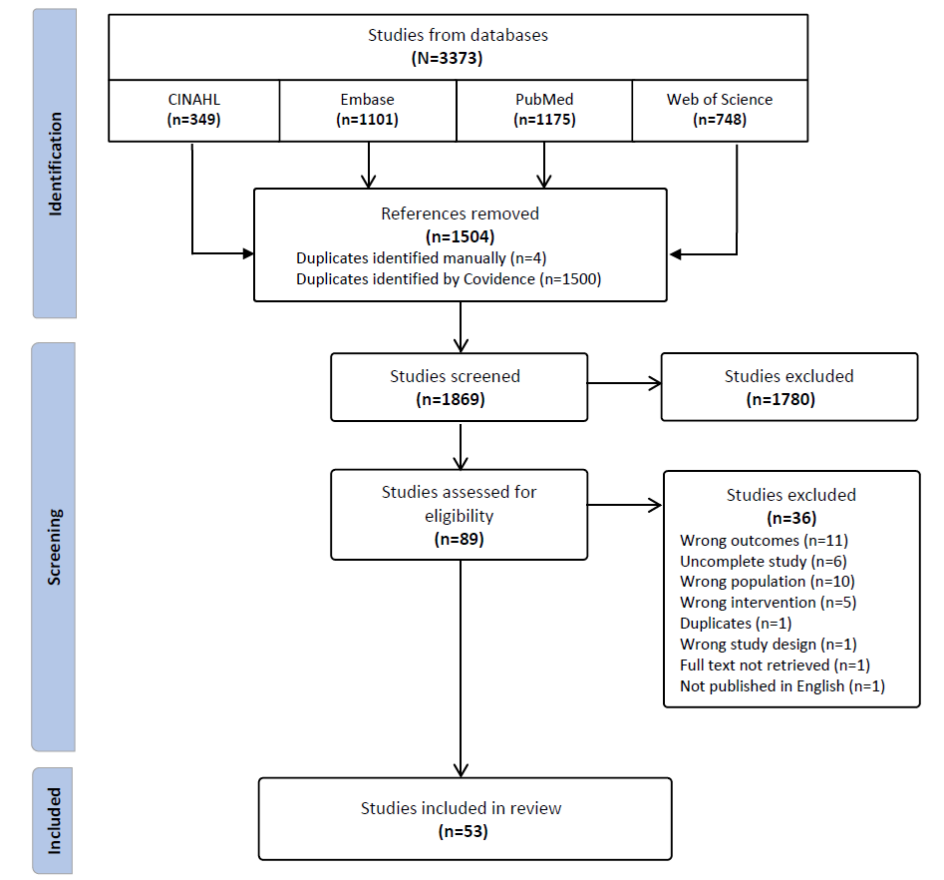
PRISMA flow diagram for study selection. PRISMA: Preferred Reporting Items for Systematic Reviews and Meta-Analyses.

In total, 3373 citations were retrieved from the database search, of which 53 (1.6%) met the inclusion criteria, encompassing a total of 34,488 participants. The number of papers at each stage and the reasons for exclusion are detailed in Figure 1.

The characteristics of the included studies are shown in Multimedia Appendix 5 [5,10,16,34-83]. Most studies were conducted in health care settings (n=35, 66%) and in high-income countries (n=49, 92.5%) [84]. RCTs were the most common research design (n=31, 58.5%). All studies had a duration of at least 3 months. The DHI duration fluctuated from 1 to 48 months. DHIs were mostly applied in combination with HCP interactions (n=43, 81.1%).

### Risk of Bias

The nonrandomized studies showed a disproportionately high number of moderate and serious risks of bias (n=17, 77.3%), predominantly due to not accurately recording and analyzing confounders. In the RCTs, the risk of bias in population health outcomes ranged from low to high, while the risks of bias in HbA_1c_ and blood glucose change outcomes exhibited some similarities, with approximately 12 of 22 (54.5%) and 10 of 23 (43.5%) studies, respectively, presenting either some concerns or high risks. However, a few studies (n=7, 77.8%) had a high risk of bias or some concerns in the T2DM development outcome. This was mainly due to discrepancies observed in the measurement of the T2DM development outcome between groups and bias resulting from missing outcome data. There were no high risks of bias in consumer experience and health care cost outcomes, with the majority having low risks (n=37, 70%, and n=35, 66.7%, respectively). The HCP experience outcome was not assessed in RCTs. There were 24 (45.3%) studies with a low risk of bias in all outcomes and 10 (41.7%) studies with both a low risk of bias and an intervention duration of at least 1 year. Details are shown in Multimedia Appendix 6 [5,10,16,34-83].

### Study Outcomes

Five outcomes were reported: consumer experience (n=13, 24.5%), health care costs (n=3, 5.7%), HCP experience (n=1, 1.9%), development of T2DM (n=14, 26.4%), and dysglycemia changes (n=52, 98.1%). Many studies reported multiple outcomes.

### Study Interventions

**Figure 2:**
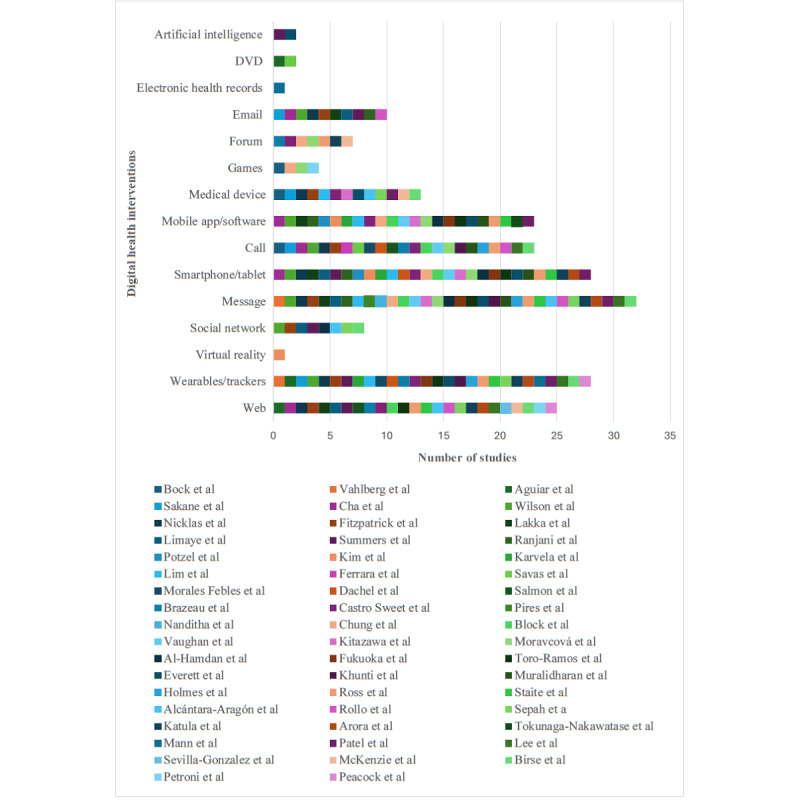
Distribution of DHIs across studies. DHI: digital health intervention.

In total, 15 DHIs were investigated. The prevalence of each DHI is visualized in Figure 2 [5,10,16,34-44,46-83,94].

Using WHO’s DHI classification, 7 DHI categories were identified: TCP, PPC, PHT, DCP, PHR, HCP DS, and TM.

For the T2DM development outcome, the effects of DHIs were positive in 1 (7.1%) study and neutral in 9 (64.3%) studies, and there were insufficient data to assess in 4 (28.6%) studies. For the dysglycemia outcome, the effects were positive in 23 (43.4%) studies and neutral in 24 (45.3%) studies, and there were insufficient data in 6 (11.3%) studies. Among the 10 (18.9%) studies with a low risk of bias in all outcomes and an intervention duration of at least 1 year, none assessed the development of T2DM; the effects of DHIs on dysglycemia were positive in 7 (70%) studies and neutral in 2 (20%) studies, and there were insufficient data in 1 (10%) study.

The effects of DHIs in all studies on consumer experience were mixed, with most being positive (n=7, 53.8%). The effects of DHIs on costs, assessed in 2 (3.8%) studies, were found to be negative. The only study reporting HCP experience indicated a positive effect.

Table 2 highlights the effect of each DHI category on each outcome. The effects of DHI categories and subcategories on the outcomes in each study are illustrated in Multimedia Appendix 7 [5,10,16,34-83].

**Table 2:** Effects of DHIs^a^ on the quadruple aims.

DHI category	Population health		Consumer experience	HCP^b^ experience	Health care costs
	T2DM^c^ development	Dysglycemia changes			
TCP^d^	Neutral/positive	Neutral/positive	Negative/neutral/positive	Positive	Negative/neutral
PPC^e^	Neutral	Neutral/positive	Negative/positive	N/A^f^	Negative
PHT^g^	Neutral/positive	Neutral/positive	Negative/neutral/positive	N/A	Negative
DCP^h^	Neutral	Neutral/positive	Neutral/positive	N/A	Negative
PHR^i^	N/A	Neutral	N/A	N/A	N/A
DS^j^	N/A	Neutral	N/A	N/A	N/A
TM^k^	Neutral/positive	Neutral/positive	Negative/neutral/positive	Positive	Negative/neutral

^a^DHI: digital health intervention.

^b^HCP: health care provider.

^c^T2DM: type 2 diabetes mellitus.

^d^TCP: targeted communication with persons.

^e^PPC: person-to-person communication.

^f^N/A: not applicable.

^g^PHT: personal health tracking.

^h^DCP: on-demand communication with persons.

^i^PHR: person-centered health records.

^j^DS: decision support.

^k^TM: telemedicine.

#### Targeted Communication With Persons

All included studies (N=53, 100%) applied TCP, including transmitting targeted health information or targeted alerts and reminders to patients. Of the 10 (18.9%) studies reviewing the effects of TCP on T2DM prevention, 9 (90%) were neutral, and 1 (10%) was positive. Of the 47 (88.7%) studies assessing dysglycemia changes, 24 (51.1%) reported neutral effects, and 23 (48.9%) posted positive effects. For example, Arora et al [34] proved after the intervention that there is a significant reduction in predicted HbA_1c_ (*P*<.001).

The effects of TCP on consumer experience was mixed, on HCP experience was positive, and on health care cost was neutral (n=1, 33.3%) or negative (n=2, 66.7%).

#### Person-to-Person Communication

Of 16 (30.2%) studies, 2 (12.5%) studies reviewing the effects of PPC on T2DM prevention were by Fitzpatrick et al [35] and McKenzie et al [36]. Results showed a neutral effect, with no significant difference in the T2DM diagnosis compared to the control group.

Of the 15 (93.8%) PPC studies assessing dysglycemia changes, 10 (66.7%) posted positive effects and 5 (33.3%) showed no discernible effect. For example, Castro Sweet et al [10] used PPC, TCP, and PHT in their DHIs; after the interventions, the change in the mean HbA_1c_ of participants reduced by 0.1% (*P*=.001).

A PPC study (6.3%) by Katula et al [37] reported consumer experience, with negative feedback of consumers.

A single study (6.3%), which was by Limaye et al [38], reported the effects of PPC on health care costs. The incremental cost of the DHI was GBP 35.8 (USD 47.5) per participant compared to GBP 23.3 (USD 30.9) per participant in the control group [38].

No study assessed the effect of PPC on HCP experience.

#### Personal Health Tracking

In 37 (69.8%) studies, PHT allowed individuals to self-monitor their health or diagnostic data (n=28, 75.7%) or actively capture/store health data to the digital platform (n=15, 40.5%).

Of the 5 (13.5%) studies on PHT reporting its effects on preventing T2DM, 1 (20%) study [39] posted a positive effect, with 4 (80%) other studies [35,36,40,41] reporting a neutral effect on T2DM development.

Of the 37 (69.8%) studies, 34 (91.9%) assessed the effectiveness of PHT in dysglycemia changes, with both positive (61.8%) and neutral (38.2%) effects. Karvela et al [42] indicated that the effect of PHT on improving HbA_1c_ was not significantly different compared to the control group (*P*=.31).

Of the 10 (27%) studies assessing the effects of PHT on consumer experience, the results varied; 4 (40%) studies showed a positive effect, 3 (30%) showed a negative effect, 2 (20%) were inconclusive or neutral, and 1 (10%) showed mixed effects. For example, Peacock et al [43] showed that participants using wearables/trackers feel empowered in their choice of appropriate foods (*P*=.04).

No studies reported HCP experience or health care costs.

#### On-Demand Communication With Persons

Of the 13 (24.5%) studies on DCP, 11 (84.6%) consisted of seeking supporting information and 3 (15.4%) simulated human-like conversations.

In addition, 2 (15.4%) studies [40,44] reported prevention effectiveness of DCP, and both had a neutral effect.

Of the 12 studies (92.3%) assessing the effects of dysglycemia, 9 (75%) reported positive population health outcomes, such as significant HbA_1c_ reduction compared to the control group in Kim et al [45] using virtual reality (VR) technology and Everett et al [46] using AI interventions (*P*<.05). Furthermore, 3 (25%) studies [40,44,47] showed the effect was neutral.

Of the 3 (23.1%) studies reporting the effectiveness of DCP on consumer experience, Cha et al [48] and Potzel et al [44] reported better experience outcomes for patients in the DHI groups, while Block et al [5] showed no effect.

Limaye et al [38] reported the effects of DCP on health care costs, with negative results. No studies reported the effects of on-demand communication on HCP experience.

#### Person-Centered Health Records and Health Care Provider Decision Support

One study applied PHR and HCP DS in their DHIs. Mann et al [49] reported no change in HbA_1c_ and blood glucose levels after the intervention. Effects on T2DM prevention, consumer experience, HCP experience, and health care costs were not included in the study.

#### Telemedicine

TM included remote consultations (n=25, 47.2%) and remote health monitoring (n=7, 13.2%).

Of the 5 (20%) studies reporting the effects of TM on T2DM development, only 1 (20%) study by Sakane et al [39] indicated that TM is effective. The other 4 (80%) studies [35,40,44,50] did not show any significant differences compared to the control group.

Of the 20 (80%) studies assessing changes in the glycemia status of participants, 11 (55%) showed that TM improves HbA_1c_ or blood glucose significantly and 9 (45%) reported neutral effects. For example, Muralidharan et al [51] and Holmes et al [52] concluded that TM, in addition to TCP, does not have a significant effect on delaying T2DM.

The effects of TM on consumer experience were varied, with 5 (71.4%) studies [39,44,48,53,54] reporting positive effects, 1 (14.2%) study [5] with no discernible effect, and 1 (14.2%) study [55] with mixed effects. For example, participants from Block et al [5] reported both positive and negative feedback.

Only 1 (14.2%) study assessed HCP experience in TM interventions, with HCP participants leaving positive feedback for TM, according to Savas et al [54].

## Discussion

### Principal Findings

This is the first systematic review to evaluate the effects of DHIs on the quadruple aims in T2DM prevention. Our findings enhance other recent studies, such as Nguyen et al [19], by offering a more comprehensive insight into the outcomes that are measured (and not measured), and the effects of DHIs on each outcome, in alignment with the quadruple aims of health care. This contributes to an evidence-based foundation for the future successful customization and implementation of DHIs for T2DM prevention. This is also the first systematic review of T2DM prevention using WHO’s DHI classification, which significantly aids in a mutually comprehensible language for various communities involved in digital health for T2DM prevention, such as technologists, researchers, clinicians, and consumers.

Our review highlights several important findings. First, there is emerging evidence supporting the effectiveness of DHIs in preventing T2DM; however, the evidence remains limited. Although only 1 study [39] reported positive effects, 9 studies indicated neutral effects and no studies reported negative effects. This is consistent with the findings by Nguyen et al [19]. Despite this, most of these neutral studies still demonstrated clinical improvements in T2DM development, though these improvements did not reach statistical significance. The evidence is clearer on dysglycemia, where the effects were positive in nearly half of the studies and neutral in the other half. Further research will need to conclusively determine the effectiveness of DHIs in preventing T2DM.

Second, the duration of DHI may play an important role in the effects on the population health outcome. All studies successful in improving dysglycemia had a DHI duration of at least 3 months, while the study successful in T2DM prevention had a duration of 12 months [39]. Among the 10 studies with a low risk of bias in all outcomes and an intervention duration of at least 1 year, a high percentage of studies (70%) demonstrated positive effects of DHIs on dysglycemia. Comparable results about dysglycemia were reported by Van Rhoon et al [18] and Donevant et al [85]. This can be because habit formation usually takes 2-3 months [86], and significant HbA_1c_ changes require at least 3 months [87]. However, recent evidence from DPPs that have proven to be successful shows that long intervention durations are required for delaying T2DM [88], with the National Health Service recommending at least 9 months and the Centers for Disease Control and Prevention suggesting 12 months. For effective T2DM prevention, a minimum DHI duration of 9-12 months may be ideal. Future research should validate these findings.

Third, there was no evidence that DHIs are effective in preventing T2DM without HCP interaction, and most studies (69.6%) successful in improving dysglycemia involved HCP interaction (75% remote, 12.5% in person, and 12.5% both). Comparable results were reported in the review by Grock et al [89], which highlighted the importance of social interaction for successful diabetes prevention interventions. The meta-analysis by Schippers et al [90] reported that mobile apps with personal interaction tools (messages, calls, email, or in-person meetings) are more effective for weight loss than automated interaction. This underscores the critical role of HCP interaction in preventing T2DM, while also revealing the promising potential of replacing in-person HCP interactions with remote interactions for effective DHIs for T2DM prevention.

Next, all studies that reported positive population health outcomes used a minimum of 2 distinct categories of DHIs. These findings share similarities with the systematic review by Van Rhoon et al [18], which suggested that interventions with a larger number of passive and interactive digital features are more effective. In our review, TCP, which involves transmitting health information or health alerts and reminders to patients, was adopted in all included studies, demonstrating its widespread use and simplicity. TCP was used in combination with other DHI categories, such as PHT, TM, PCP, or DCP.

PHT showed evidence as a potentially useful tool for T2DM prevention and dysglycemia improvement in our review. Similarly, many studies showed that PHT successfully increases physical activity and decreases a sedentary lifestyle [91-93]. The effect of PHT on consumer experience was mixed. Automatic data capture in wearables and medical devices received more positive feedback than manual data capture, which had only neutral or negative effects. This is likely because data capture is more aligned with manual tasks, whereas wearables and medical devices are designed for automatic data collection. This result aligns with the study by Kim et al [94] on self-tracking via a web-based platform. Participants using devices with automatic data entry engaged with the platform 4 times longer than those who manually entered data [94]. This evidence strongly suggests that PHT, particularly automated tracking devices, could play a pivotal role in the prevention strategies for T2DM.

Our review suggests that TM may be effective in preventing T2DM and managing dysglycemia. This aligns with the review by Nguyen et al [19]. TM consists of remote consultations through calls and messages, and remote health monitoring. This monitoring can be achieved either automatically or manually via PHT tools, such as wearables, medical devices, or web-based apps. Consequently, there is a significant correlation between TM and PHT. Our review also shows evidence that the combination of TM, PHT, and TCP is effective in T2DM prevention. This suggests that such DHIs should not only be embraced but also be integrated with other DHIs in T2DM prevention.

Although PPC or DCP did not prove effective in preventing T2DM, they showed evidence of improving dysglycemia. PPC may be beneficial in glycemic control for diabetes management [95,96].

There is evidence that using DCP (look-up tools, human-like conversations) is helpful in diabetes prevention and management. In our review, AI used with wearables showed positive effects on glycemic status [46,56]. Similarly, evidence indicates that using AI technology in diabetes management is effective when combined with wearable technologies [97]. Advanced algorithms and data from everyday participants’ activities allowed AI to provide lifestyle recommendations, which were real time, personalized, and contextual for each participant, contributing to delivering patient-centered care [98]. VR was used in 1 study, providing an immersive experience that increased participant engagement and enjoyment [99]. This highlights an advantage of VR technology. Further studies are needed to explore the potential of PPC, AI, and VR in T2DM prevention.

Health records and HCP DS were the least common in our review, with only 1 included study showing neutral effects. These DHIs target HCPs rather than consumers. Since preventing T2DM requires participants to modify lifestyles for a long period, DHIs that motivate and engage consumers may be more beneficial than DHIs targeting HCPs alone. Further studies should explore combining these DHIs with consumer-targeted interventions.

Finally, there were insufficient studies assessing HCP experience (1 study) and health care costs (3 studies). For other topics, there were several studies focusing on the effects of DHIs on HCP experience. Lampickienė et al [100] concluded that HCPs mostly report positive experiences with digital consultations, which have advantages for HCPs and patients. Studies that reported health care costs of DHIs were sparse; all 3 studies in our review indicated no positive cost outcomes in DHIs. According to a systematic review of DHIs by Gentili et al [101], there is convincing evidence of the cost-effectiveness of DHI in health care. They indicated that several types of DHIs, such as videoconferencing systems, messaging, calls, mobile apps, and web-based platforms, help reduce health care costs. Although the findings in our review were different, the small number of studies suggests that it may still be feasible to implement DHIs that are cost-effective in T2DM prevention. Further studies implementing DHIs should assess not only population health outcomes and consumer experiences but also HCP experiences and health care costs.

### Limitations

There are some limitations of our review. The diagnosis criteria for T2DM varied slightly across studies. This is because of different guidelines, resources, and clinical considerations. Although our study reflected a real-world scenario, the variation in diagnosis criteria could potentially lead to data inconsistencies, because different diagnosis criteria may classify the same participant differently.

The inclusion criteria of our study permitted a wide range of variability in aspects, such as study design, study population, DHIs, and duration. DHIs are intended for real-life implementation, and the condition of RCTs is unlikely to match those routine settings, while before-and-after studies or cohort studies can provide valuable insights into special populations, such as patients with chronic liver disease [57] or renal transplant [58], groups that are often difficult to enroll in RCTs. This diversity, while inclusive, posed challenges in drawing direct comparisons and made it unfeasible to conduct a meta-analysis or certainty-of-evidence assessment.

The intervention duration fluctuated between 1 month and 48 months. Chronic diseases, such as T2DM, develop over a long period [102]. Short-term studies may not allow for the comprehensive effects of DHIs to show a clear impact on disease progression. More studies with longer durations are needed. Our study did not summarize the intensity and frequency of the DHI used in each included study. To provide a more comprehensive understanding of the effects of DHIs, further reviews and studies should examine the intensity and frequency of DHIs, rather than solely focusing on their duration.

Finally, our inclusion criteria included papers in English only. This may have potentially excluded some relevant studies.

### Conclusion

The findings from this systematic review demonstrate that the effects of DHIs on the quadruple aims in T2DM prevention have proven benefits for population health, mixed results for consumer experience, and insufficient studies on HCP experience and health care costs. Further studies should prioritize improving consumer experience, while also addressing HCP experience and health care costs.

Although evidence supporting the effectiveness of DHIs in reducing the burden of T2DM remains limited, it is clear DHIs are effective in improving dysglycemia. To maximize their effectiveness in preventing T2DM and managing dysglycemia, DHIs should be strategically integrated with in-person or remote HCP interaction. The incorporation of health information transmission, alerts, and reminders for targeted individuals, along with TM and PHT strategies, is paramount. Peer group support, look-up tools, AI, and VR hold promising potential for future exploration in this field. We anticipate the advancement in these technologies will significantly influence the prevention of T2DM in the future.

## Appendix

Multimedia Appendix 1 PRISMA 2020 checklist. PRISMA: Preferred Reporting Items for Systematic Reviews and Meta-Analyses.

Multimedia Appendix 2 Search string.

Multimedia Appendix 3 Data extraction form.

Multimedia Appendix 4 WHO’s DHI classification. DHI: digital health intervention; WHO: World Health Organization.

Multimedia Appendix 5 Study characteristics.

Multimedia Appendix 6 Risk assessment.

Multimedia Appendix 7 Effects of DHIs on the quadruple aims. DHI: digital health intervention.

## Abbreviations

AI: artificial intelligence
DCP: on-demand communication with persons
DHI: Digital health intervention
DPP: diabetes prevention program
DS: decision support
HbA_1c_: hemoglobin A_1c_
HCP: health care provider
PHR: person-centered health records
PHT: personal health tracking
PPC: person-to-person communication
PRISMA: Preferred Reporting Items for Systematic Reviews and Meta-Analyses
RCT: randomized controlled trial
RoB 2: risk-of-bias version 2
ROBINS-I: risk of bias in nonrandomized studies of interventions
T2DM: type 2 diabetes mellitus
TCP: targeted communication with persons
TM: telemedicine
VR: virtual reality
WHO: World Health Organization

## Data Availability

All data generated and analyzed during this study are included in the published paper (and its supplementary files).

## References

[1] Magliano D, Boyko E (2021). IDF Diabetes Atlas.

[2] Alberti KGMM, Zimmet P, Shaw J (2007). International Diabetes Federation: a consensus on type 2 diabetes prevention. Diabet Med.

[3] Ramachandran A, Snehalatha C, Mary S, Mukesh B, Bhaskar AD, Vijay V, Indian Diabetes Prevention Programme (IDPP) (2006). The Indian Diabetes Prevention Programme shows that lifestyle modification and metformin prevent type 2 diabetes in Asian Indian subjects with impaired glucose tolerance (IDPP-1). Diabetologia.

[4] Tuomilehto J, Lindström J, Eriksson JG, Valle TT, Hämäläinen H, Ilanne-Parikka P, Keinänen-Kiukaanniemi S, Laakso M, Louheranta A, Rastas M, Salminen V, Uusitupa M, Finnish Diabetes Prevention Study Group (2001). Prevention of type 2 diabetes mellitus by changes in lifestyle among subjects with impaired glucose tolerance. N Engl J Med.

[5] Block G, Azar KM, Romanelli RJ, Block TJ, Hopkins D, Carpenter HA, Dolginsky MS, Hudes ML, Palaniappan LP, Block CH (2015). Diabetes prevention and weight loss with a fully automated behavioral intervention by email, web, and mobile phone: a randomized controlled trial among persons with prediabetes. J Med Internet Res.

[6] ElSayed NA, Aleppo G, Aroda VR, Bannuru RR, Brown FM, Bruemmer D, Collins BS, Hilliard ME, Isaacs D, Johnson EL, Kahan S, Khunti K, Leon J, Lyons SK, Perry ML, Prahalad P, Pratley RE, Seley JJ, Stanton RC, Gabbay RA, on behalf of the American Diabetes Association (2023). 3. Prevention or delay of type 2 diabetes and associated comorbidities: standards of care in diabetes-2023. Diabetes Care.

[7] World Health Organization (2023). Classification of Digital Interventions, Services and Applications in Health: A Shared Language to Describe the Uses of Digital Technology for Health (2nd Ed.).

[8] Hambleton SJ, Aloizos AMJ (2019). Australia's digital health journey. Med J Aust.

[9] World Health Organization (2018). Classification of Digital Health Interventions v 1.

[10] Castro Sweet CM, Chiguluri V, Gumpina R, Abbott P, Madero EN, Payne M, Happe L, Matanich R, Renda A, Prewitt T (2018). Outcomes of a digital health program with human coaching for diabetes risk reduction in a Medicare population. J Aging Health.

[11] Sly B, Russell AW, Sullivan C (2022). Digital interventions to improve safety and quality of inpatient diabetes management: a systematic review. Int J Med Inform.

[12] Ehrhardt N, Al Zaghal E (2019). Behavior modification in prediabetes and diabetes: potential use of real-time continuous glucose monitoring. J Diabetes Sci Technol.

[13] Kario K, Harada N, Okura A (2022). Digital therapeutics in hypertension: evidence and perspectives. Hypertension.

[14] Shah N, Costello K, Mehta A, Kumar D (2022). Applications of digital health technologies in knee osteoarthritis: narrative review. JMIR Rehabil Assist Technol.

[15] Janjua S, Banchoff E, Threapleton CJ, Prigmore S, Fletcher J, Disler RT (2021). Digital interventions for the management of chronic obstructive pulmonary disease. Cochrane Database Syst Rev.

[16] Toro-Ramos T, Michaelides A, Anton M, Karim Z, Kang-Oh L, Argyrou C, Loukaidou E, Charitou MM, Sze W, Miller JD (2020). Mobile delivery of the diabetes prevention program in people with prediabetes: randomized controlled trial. JMIR Mhealth Uhealth.

[17] Singareddy S, Sn V, Jaramillo A, Yasir M, Iyer N, Hussein S, Nath TS (2023). Artificial intelligence and its role in the management of chronic medical conditions: a systematic review. Cureus.

[18] Van Rhoon L, Byrne M, Morrissey E, Murphy J, McSharry J (2020). A systematic review of the behaviour change techniques and digital features in technology-driven type 2 diabetes prevention interventions. Digit Health.

[19] Nguyen V, Ara P, Simmons D, Osuagwu UL (2024). The role of digital health technology interventions in the prevention of type 2 diabetes mellitus: a systematic review. Clin Med Insights Endocrinol Diabetes.

[20] Bodenheimer T, Sinsky C (2014). From triple to quadruple aim: care of the patient requires care of the provider. Ann Fam Med.

[21] Leal Neto O, Von Wyl V (2024). Digital transformation of public health for noncommunicable diseases: narrative viewpoint of challenges and opportunities. JMIR Public Health Surveill.

[22] Mattison G, Canfell O, Forrester D, Dobbins C, Smith D, Töyräs J, Sullivan C (2022). The influence of wearables on health care outcomes in chronic disease: systematic review. J Med Internet Res.

[23] Bhatti S, Dahrouge S, Muldoon L, Rayner J (2022). Using the quadruple aim to understand the impact of virtual delivery of care within Ontario community health centres: a qualitative study. BJGP Open.

[24] Asthana S, Prime S (2023). The role of digital transformation in addressing health inequalities in coastal communities: barriers and enablers. Front Health Serv.

[25] Woods L, Eden R, Canfell OJ, Nguyen K, Comans T, Sullivan C (2023). Show me the money: how do we justify spending health care dollars on digital health?. Med J Aust.

[26] Laur C, Agarwal P, Thai K, Kishimoto V, Kelly S, Liang K, Bhatia RS, Bhattacharyya O, Martin D, Mukerji G (2022). Implementation and evaluation of COVIDCare@Home, a family medicine-led remote monitoring program for patients with COVID-19: multimethod cross-sectional study. JMIR Hum Factors.

[27] Page MJ, McKenzie JE, Bossuyt PM, Boutron I, Hoffmann TC, Mulrow CD, Shamseer L, Tetzlaff JM, Akl EA, Brennan SE, Chou R, Glanville J, Grimshaw JM, Hróbjartsson A, Lalu MM, Li T, Loder EW, Mayo-Wilson E, McDonald S, McGuinness LA, Stewart LA, Thomas J, Tricco AC, Welch VA, Whiting P, Moher D (2021). The PRISMA 2020 statement: an updated guideline for reporting systematic reviews. BMJ.

[28] Wolf JA, Niederhauser V, Marshburn D, LaVela SL (2014). Defining patient experience. Patient Experience J.

[29] Benson T, Benson A (2023). Routine measurement of patient experience. BMJ Open Qual.

[30] (2023). New model of employee experience can help organizations drive growth, retention and resilience Internet. World Economic Forum.

[31] Neri S, Ornaghi A, Michalos AC (2014). Health-care costs. Encyclopedia of Quality of Life and Well-Being Research.

[32] Sterne JAC, Savović J, Page MJ, Elbers RG, Blencowe NS, Boutron I, Cates CJ, Cheng H, Corbett MS, Eldridge SM, Emberson JR, Hernán MA, Hopewell S, Hróbjartsson A, Junqueira DR, Jüni P, Kirkham JJ, Lasserson T, Li T, McAleenan A, Reeves BC, Shepperd S, Shrier I, Stewart LA, Tilling K, White IR, Whiting PF, Higgins JPT (2019). RoB 2: a revised tool for assessing risk of bias in randomised trials. BMJ.

[33] Sterne J, Hernán MA, Reeves B, Savović J, Berkman N, Viswanathan M, Henry D, Altman DG, Ansari MT, Boutron I, Carpenter JR, Chan A-W, Churchill R, Deeks JJ, Hróbjartsson A, Kirkham J, Jüni P, Loke YK, Pigott TD, Ramsay CR, Regidor D, Rothstein HR, Sandhu L, Santaguida PL, Schünemann HJ, Shea B, Shrier I, Tugwell P, Turner L, Valentine JC, Waddington H, Waters E, Wells GA, Whiting PF, Higgins JPt (2016). ROBINS-I: a tool for assessing risk of bias in non-randomised studies of interventions. BMJ.

[34] Arora S, Lam CN, Burner E, Menchine M (2024). Implementation and evaluation of an automated text message-based diabetes prevention program for adults with pre-diabetes. J Diabetes Sci Technol.

[35] Fitzpatrick S, Mayhew M, Rawlings A, Smith N, Nyongesa D, Vollmer W, Stevens VJ, Grall SK, Fortmann SP (2022). Evaluating the implementation of a digital diabetes prevention program in an integrated health care delivery system among older adults: results of a natural experiment. Clin Diabetes.

[36] McKenzie AL, Athinarayanan SJ, McCue JJ, Adams RN, Keyes M, McCarter JP, Volek JS, Phinney SD, Hallberg SJ (2021). Type 2 diabetes prevention focused on normalization of glycemia: a two-year pilot study. Nutrients.

[37] Katula JA, Dressler EV, Kittel CA, Harvin LN, Almeida FA, Wilson KE, Michaud TL, Porter GC, Brito FA, Goessl CL, Jasik CB, Sweet CMC, Schwab R, Estabrooks PA (2022). Effects of a digital diabetes prevention program: an RCT. Am J Prev Med.

[38] Limaye T, Kumaran K, Joglekar C, Bhat D, Kulkarni R, Nanivadekar A, Yajnik C (2017). Efficacy of a virtual assistance-based lifestyle intervention in reducing risk factors for type 2 diabetes in young employees in the information technology industry in India: LIMIT, a randomized controlled trial. Diabet Med.

[39] Sakane N, Kotani K, Takahashi K, Sano Y, Tsuzaki K, Okazaki K, Sato J, Suzuki S, Morita S, Oshima Y, Izumi K, Kato M, Ishizuka N, Noda M, Kuzuya H (2015). Effects of telephone-delivered lifestyle support on the development of diabetes in participants at high risk of type 2 diabetes: J-DOIT1, a pragmatic cluster randomised trial. BMJ Open.

[40] Nicklas JM, Zera CA, England LJ, Rosner BA, Horton E, Levkoff SE, Seely EW (2014). A web-based lifestyle intervention for women with recent gestational diabetes mellitus: a randomized controlled trial. Obstet Gynecol.

[41] Staite E, Bayley A, Al-Ozairi E, Stewart K, Hopkins D, Rundle J, Basudev N, Mohamedali Z, Ismail K (2020). A wearable technology delivering a web-based diabetes prevention program to people at high risk of type 2 diabetes: randomized controlled trial. JMIR Mhealth Uhealth.

[42] Karvela M, Golden CT, Bell N, Martin-Li S, Bedzo-Nutakor J, Bosnic N, DeBeaudrap P, de Mateo-Lopez S, Alajrami A, Qin Y, Eze M, Hon T, Simón-Sánchez J, Sahoo R, Pearson-Stuttard J, Soon-Shiong P, Toumazou C, Oliver N (2024). Assessment of the impact of a personalised nutrition intervention in impaired glucose regulation over 26 weeks: a randomised controlled trial. Sci Rep.

[43] Peacock AS, Bogossian FE, Wilkinson SA, Gibbons KS, Kim C, McIntyre HD (2015). A randomised controlled trial to delay or prevent type 2 diabetes after gestational diabetes: walking for exercise and nutrition to prevent diabetes for you. Int J Endocrinol.

[44] Potzel AL, Gar C, Banning F, Sacco V, Fritsche A, Fritsche L, Müssig K, Dauben L, Seissler J, Lechner A (2022). A novel smartphone app to change risk behaviors of women after gestational diabetes: a randomized controlled trial. PLoS One.

[45] Kim S, Kim HJ, Shin G (2021). Self-management mobile virtual reality program for women with gestational diabetes. Int J Environ Res Public Health.

[46] Everett E, Kane B, Yoo A, Dobs A, Mathioudakis N (2018). A novel approach for fully automated, personalized health coaching for adults with prediabetes: pilot clinical trial. J Med Internet Res.

[47] Chung H, Tai C, Chang P, Su W, Chien L (2023). The effectiveness of a traditional Chinese medicine-based mobile health app for individuals with prediabetes: randomized controlled trial. JMIR Mhealth Uhealth.

[48] Cha E, Kim KH, Umpierrez G, Dawkins CR, Bello MK, Lerner HM, Narayan KMV, Dunbar SB (2014). A feasibility study to develop a diabetes prevention program for young adults with prediabetes by using digital platforms and a handheld device. Diabetes Educ.

[49] Mann DM, Palmisano J, Lin JJ (2016). A pilot randomized trial of technology-assisted goal setting to improve physical activity among primary care patients with prediabetes. Prev Med Rep.

[50] Ferrara A, Hedderson M, Brown S, Albright C, Ehrlich S, Tsai A, Caan BJ, Sternfeld B, Gordon NP, Schmittdiel JA, Gunderson EP, Mevi AA, Herman WH, Ching J, Crites Y, Quesenberry CP (2016). The comparative effectiveness of diabetes prevention strategies to reduce postpartum weight retention in women with gestational diabetes mellitus: the Gestational Diabetes' Effects on Moms (GEM) cluster randomized controlled trial. Diabetes Care.

[51] Muralidharan S, Ranjani H, Anjana RM, Gupta Y, Ambekar S, Koppikar V, Jagannathan N, Jena S, Tandon N, Allender S, Mohan V (2021). Change in cardiometabolic risk factors among Asian Indian adults recruited in a mHealth-based diabetes prevention trial. Digit Health.

[52] Holmes VA, Draffin CR, Patterson CC, Francis L, Irwin J, McConnell M, Farrell B, Brennan SF, McSorley O, Wotherspoon AC, Davies M, McCance DR, PAIGE Study Group (2018). Postnatal lifestyle intervention for overweight women with previous gestational diabetes: a randomized controlled trial. J Clin Endocrinol Metab.

[53] Bock BC, Dunsiger SI, Wu W, Ciccolo JT, Serber ER, Lantini R, Marcus BH (2019). Reduction in HbA1c with exercise videogames among participants with elevated HbA1c: secondary analysis of the Wii Heart Fitness trial. Diabetes Res Clin Pract.

[54] Savas L, Grady K, Cotterill S, Summers L, Boaden R, Gibson J (2015). Prioritising prevention: implementation of IGT Care Call, a telephone based service for people at risk of developing type 2 diabetes. Prim Care Diabetes.

[55] Dachel TA, Mota D (2021). Technology and human connection to prevent diabetes in rural United States. J Nurse Pract.

[56] Summers C, Tobin S, Unwin D (2021). Evaluation of the low carb program digital intervention for the self-management of type 2 diabetes and prediabetes in an NHS England general practice: single-arm prospective study. JMIR Diabetes.

[57] Petroni ML, Brodosi L, Armandi A, Marchignoli F, Bugianesi E, Marchesini G (2023). Lifestyle intervention in NAFLD: long-term diabetes incidence in subjects treated by web- and group-based programs. Nutrients.

[58] Morales Febles R, Marrero Miranda D, Jiménez Sosa A, González Rinne A, Cruz Perera C, Rodríguez-Rodríguez AE, Álvarez González A, Díaz Martín L, Negrín Mena N, Acosta Sørensen C, Pérez Tamajón L, Rodríguez Hernández A, González Rinne F, Dorta González A, Ledesma Pérez E, González Delgado A, Domínguez-Rodríguez A, García Baute MDC, Torres Ramírez A, Porrini E (2023). Exercise and prediabetes after renal transplantation (EXPRED-I): a prospective study. Sports Med Open.

[59] Aguiar EJ, Morgan PJ, Collins CE, Plotnikoff RC, Young MD, Callister R (2016). Efficacy of the type 2 diabetes prevention using lifestyle education program RCT. Am J Prev Med.

[60] Alcántara-Aragón V, Rodrigo-Cano S, Lupianez-Barbero A, Martinez M, Martinez C, Tapia J, Iniesta JM, Tenes S, Urgell E, Navarro G, Hernando ME, Merino-Torres JF, de Leiva A, Gonzalez C (2018). Web support for weight-loss interventions: PREDIRCAM2 clinical trial baseline characteristics and preliminary results. Diabetes Technol Ther.

[61] Al-Hamdan R, Avery A, Al-Disi D, Sabico S, Al-Daghri NM, McCullough F (2021). Efficacy of lifestyle intervention program for Arab women with prediabetes using social media as an alternative platform of delivery. J Diabetes Investig.

[62] Birse CE, McPhaul MJ, Arellano AR, Fragala MS, Lagier RJ (2022). Impact of a digital diabetes prevention program on estimated 8-year risk of diabetes in a workforce population. J Occup Environ Med.

[63] Brazeau A, Leong A, Meltzer SJ, Cruz R, DaCosta D, Hendrickson-Nelson M, Joseph L, Dasgupta K, MoMM study group (2014). Group-based activities with on-site childcare and online support improve glucose tolerance in women within 5 years of gestational diabetes pregnancy. Cardiovasc Diabetol.

[64] Fukuoka Y, Gay C, Joiner K, Vittinghoff E (2015). A novel mobile phone delivered diabetes prevention program in overweight adults at risk for type 2 diabetes - a randomized controlled trial. Am J Prev Med.

[65] Khunti K, Griffin S, Brennan A, Dallosso H, Davies M, Eborall H, Edwardson C, Gray L, Hardeman W, Heathcote L, Henson J, Morton K, Pollard D, Sharp S, Sutton S, Troughton J, Yates T (2021). Behavioural interventions to promote physical activity in a multiethnic population at high risk of diabetes: PROPELS three-arm RCT. Health Technol Assess.

[66] Kitazawa M, Takeda Y, Hatta M, Horikawa C, Sato T, Osawa T, Ishizawa M, Suzuki H, Matsubayashi Y, Fujihara K, Yamada T, Sone H (2024). Lifestyle intervention with smartphone app and isCGM for people at high risk of type 2 diabetes: randomized trial. J Clin Endocrinol Metab.

[67] Lakka TA, Aittola K, Järvelä-Reijonen E, Tilles-Tirkkonen T, Männikkö R, Lintu N, Karhunen L, Kolehmainen M, Harjumaa M, Mattila E, Järvenpää R, Ermes M, Mikkonen S, Martikainen J, Poutanen K, Schwab U, Absetz P, Lindström J, Pihlajamäki J (2023). Real-world effectiveness of digital and group-based lifestyle interventions as compared with usual care to reduce type 2 diabetes risk - a stop diabetes pragmatic randomised trial. Lancet Reg Health Eur.

[68] Lee J, Lim S, Cha S, Han C, Jung AR, Kim K, Yoon K, Ko S (2021). Short-term effects of the internet-based Korea Diabetes Prevention Study: 6-month results of a community-based randomized controlled trial. Diabetes Metab J.

[69] Lim S, Ong K, Johal J, Han C, Yap Q, Chan Y, Zhang ZP, Chandra CC, Thiagarajah AG, Khoo CM (2021). A smartphone app-based lifestyle change program for prediabetes (D'LITE Study) in a multiethnic Asian population: a randomized controlled trial. Front Nutr.

[70] Moravcová K, Karbanová M, Bretschneider MP, Sovová M, Ožana J, Sovová E (2022). Comparing digital therapeutic intervention with an intensive obesity management program: randomized controlled trial. Nutrients.

[71] Nanditha A, Thomson H, Susairaj P, Srivanichakorn W, Oliver N, Godsland IF, Majeed A, Darzi A, Satheesh K, Simon M, Raghavan A, Vinitha R, Snehalatha C, Westgate K, Brage S, Sharp SJ, Wareham NJ, Johnston DG, Ramachandran A (2020). A pragmatic and scalable strategy using mobile technology to promote sustained lifestyle changes to prevent type 2 diabetes in India and the UK: a randomised controlled trial. Diabetologia.

[72] Pires M, Shaha S, King C, Morrison J, Nahar T, Ahmed N, Jennings HM, Akter K, Haghparast-Bidgoli H, Khan AKA, Costello A, Kuddus A, Azad K, Fottrell E (2022). Equity impact of participatory learning and action community mobilisation and mHealth interventions to prevent and control type 2 diabetes and intermediate hyperglycaemia in rural Bangladesh: analysis of a cluster randomised controlled trial. J Epidemiol Community Health.

[73] Ranjani H, Nitika S, Anjana R, Ramalingam S, Mohan V, Saligram N (2020). Impact of noncommunicable disease text messages delivered via an app in preventing and managing lifestyle diseases: results of the "myArogya" worksite-based effectiveness study from India. J Diabetol.

[74] Rollo ME, Baldwin JN, Hutchesson M, Aguiar EJ, Wynne K, Young A, Callister R, Haslam R, Collins CE (2020). The feasibility and preliminary efficacy of an eHealth lifestyle program in women with recent gestational diabetes mellitus: a pilot study. Int J Environ Res Public Health.

[75] Ross JAD, Barron E, McGough B, Valabhji J, Daff K, Irwin J, Henley WE, Murray E (2022). Uptake and impact of the English National Health Service digital diabetes prevention programme: observational study. BMJ Open Diabetes Res Care.

[76] Salmon MK, Gordon NF, Constantinou D, Reid KS, Wright BS, Kridl TL, Faircloth GC (2022). Comparative effectiveness of lifestyle intervention on fasting plasma glucose in normal weight versus overweight and obese adults with prediabetes. Am J Lifestyle Med.

[77] Sepah SC, Jiang L, Peters AL (2015). Long-term outcomes of a web-based diabetes prevention program: 2-year results of a single-arm longitudinal study. J Med Internet Res.

[78] Sevilla-Gonzalez MDR, Bourguet-Ramirez B, Lazaro-Carrera LS, Martagon-Rosado AJ, Gomez-Velasco DV, Viveros-Ruiz TL (2022). Evaluation of a web platform to record lifestyle habits in subjects at risk of developing type 2 diabetes in a middle-income population: prospective interventional study. JMIR Diabetes.

[79] Tokunaga-Nakawatase Y, Nishigaki M, Taru C, Miyawaki I, Nishida J, Kosaka S, Sanada H, Kazuma K (2014). Computer-supported indirect-form lifestyle-modification support program using Lifestyle Intervention Support Software for Diabetes Prevention (LISS-DP) for people with a family history of type 2 diabetes in a medical checkup setting: a randomized controlled trial. Prim Care Diabetes.

[80] Vahlberg B, Lundström E, Eriksson S, Holmback U, Cederholm T (2021). Potential effects on cardiometabolic risk factors and body composition by short message service (SMS)-guided training after recent minor stroke or transient ischaemic attack: post hoc analyses of the STROKEWALK randomised controlled trial. BMJ Open.

[81] Vaughan EM, Cardenas VJ, Chan W, Amspoker AB, Johnston CA, Virani SS, Ballantyne CM, Naik AD (2024). Implementation and evaluation of a mHealth-based community health worker feedback loop for Hispanics with and at risk for diabetes. J Gen Intern Med.

[82] Wilson MG, Castro Sweet CM, Edge MD, Madero EN, McGuire M, Pilsmaker M, Carpenter D, Kirschner S (2017). Evaluation of a digital behavioral counseling program for reducing risk factors for chronic disease in a workforce. J Occup Environ Med.

[83] Patel MS, Polsky D, Small DS, Park S, Evans CN, Harrington T, Djaraher R, Changolkar S, Snider CK, Volpp KG (2021). Predicting changes in glycemic control among adults with prediabetes from activity patterns collected by wearable devices. NPJ Digit Med.

[84] (2024). World Bank country and lending groups. World Bank.

[85] Donevant SB, Estrada RD, Culley JM, Habing B, Adams SA (2018). Exploring app features with outcomes in mHealth studies involving chronic respiratory diseases, diabetes, and hypertension: a targeted exploration of the literature. J Am Med Inform Assoc.

[86] Gardner B, Lally P, Wardle J (2012). Making health habitual: the psychology of 'habit-formation' and general practice. Br J Gen Pract.

[87] ElSayed NA, Aleppo G, Aroda VR, Bannuru RR, Brown FM, Bruemmer D, Collins BS, Hilliard ME, Isaacs D, Johnson EL, Kahan S, Khunti K, Leon J, Lyons SK, Perry ML, Prahalad P, Pratley RE, Seley JJ, Stanton RC, Gabbay RA, on behalf of the American Diabetes Association (2023). 6. Glycemic targets: standards of care in diabetes-2023. Diabetes Care.

[88] Diabetes prevention program (DPP). National Institutes of Health.

[89] Grock S, Ku J, Kim J, Moin T (2017). A review of technology-assisted interventions for diabetes prevention. Curr Diabetes Rep.

[90] Schippers M, Adam PCG, Smolenski DJ, Wong HTH, de Wit JBF (2017). A meta-analysis of overall effects of weight loss interventions delivered via mobile phones and effect size differences according to delivery mode, personal contact, and intervention intensity and duration. Obes Rev.

[91] Barwais FA, Cuddihy TF, Tomson LM (2013). Physical activity, sedentary behavior and total wellness changes among sedentary adults: a 4-week randomized controlled trial. Health Qual Life Outcomes.

[92] Hartz J, Yingling L, Powell-Wiley TM (2016). Use of mobile health technology in the prevention and management of diabetes mellitus. Curr Cardiol Rep.

[93] Finkelstein J, Bedra M, Li X, Wood J, Ouyang P (2015). Mobile app to reduce inactivity in sedentary overweight women. Stud Health Technol Inform.

[94] Kim JY, Wineinger NE, Taitel M, Radin JM, Akinbosoye O, Jiang J, Nikzad N, Orr G, Topol E, Steinhubl S (2016). Self-monitoring utilization patterns among individuals in an incentivized program for healthy behaviors. J Med Internet Res.

[95] Werner JJ, Ufholz K, Yamajala P (2024). Recent findings on the effectiveness of peer support for patients with type 2 diabetes. Curr Cardiovasc Risk Rep.

[96] Tang PY, Duni J, Peeples MM, Kowitt SD, Bhushan NL, Sokol RL, Fisher EB (2021). Complementarity of digital health and peer support: "this is what's coming". Front Clin Diabetes Healthc.

[97] Makroum MA, Adda M, Bouzouane A, Ibrahim H (2022). Machine learning and smart devices for diabetes management: systematic review. Sensors (Basel).

[98] World Health Organization (2016). WHO Global Strategy on Integrated People-Centred Health Services 2016-2026.

[99] Dương Tq, Soldera J (2024). Virtual reality tools for training in gastrointestinal endoscopy: a systematic review. Artif Intell Gastrointest Endosc.

[100] Lampickienė I, Davoody N (2022). Healthcare professionals' experience of performing digital care visits-a scoping review. Life (Basel).

[101] Gentili A, Failla G, Melnyk A, Puleo V, Tanna GLD, Ricciardi W, Cascini F (2022). The cost-effectiveness of digital health interventions: a systematic review of the literature. Front Public Health.

[102] American Diabetes Association Professional Practice Committee (2021). 2. Classification and diagnosis of diabetes: standards of medical care in diabetes. Diabetes Care.

