# Supplementary material for: Digital Health Interventions to Prevent Type 2 Diabetes Mellitus: Systematic Review
Source: J Med Internet Res. 2025 Apr 25;27:e67507. doi: 10.2196/67507 (PMC12064978; doi:10.2196/67507)
Supplement: Multimedia Appendix 2 [file jmir_v27i1e67507_app2.pdf]

Multimedia Appendix 2. Search string

PubMed

("Digital Health"[Mesh] OR "Telemedicine"[Mesh] OR "Wearable Electronic Devices"[Mesh] OR "Mobile Applications"[Mesh] OR "Smartphone"[Mesh] OR “Artificial Intelligence"[Mesh] OR “Machine Learning"[Mesh] OR *"Fitness Trackers"[Mesh]* OR "Social Media"[Mesh] OR "Video Games"[Mesh] OR "Virtual Reality"[Mesh] OR "Internet"[Mesh]

OR "digital technology"[tiab] OR "digital tool"[tiab] OR "digital health"[tiab] OR “digital platform*”[tiab] OR "artificial intelligence"[tiab] OR AI[tiab] OR “machine learning”[tiab] OR “federated learning”[tiab] OR “computational intelligence”[tiab] OR “machine intelligence”[tiab] OR "computer reasoning"[tiab] OR “big data”[tiab] OR "deep learning"[tiab] OR "user-computer interface"[tiab] OR tablet[tiab] OR iPad[tiab] OR “Smartphone”[tiab] OR “Smart phone”[tiab] OR “Smartwatch”[tiab] OR "portable game"[tiab] OR "video games"[tiab] OR "digital assistant"[tiab] OR “Digital Therapeutics”[tiab] OR "social media"[tiab] OR "clinical decision support systems"[tiab] OR "clinical decision support system"[tiab] OR "decision support system"[tiab] OR "decision support systems"[tiab] OR eHealth[tiab] OR "electronic health"[tiab] OR e-health[tiab] OR "Telemedicine"[tiab] OR "Tele-medicine"[tiab] OR "Tele medicine"[tiab] OR “mHealth”[tiab] OR “m-Health”[tiab] OR “m Health”[tiab] OR “Mobile Health”[tiab] OR “MobileHealth”[tiab] OR “Mobile-Health”[tiab] OR "mobile technology"[tiab] OR "mobile applications"[tiab] OR "mobile application"[tiab] OR “mobile app”[tiab] OR "mobile device"[tiab] OR “eHealth”[tiab] OR “e-Health”[tiab] OR “e Health”[tiab] OR “Telehealth”[tiab] OR “Tele health”[tiab] OR “Tele-health”[tiab] OR “Tele monitor*”[tiab] OR “Tele-monitor*”[tiab] OR “Telemonitor*”[tiab] OR “Wearable Electronic Device*”[tiab] OR “Wearable Device*”[tiab] OR “Health Tracking Devices”[tiab] OR “wireless device*”[tiab] OR “Mobile Phone”[tiab] OR “MobilePhone”[tiab] OR “Mobile Telephone”[tiab]

OR “pedometer*”[tiab] OR “virtual reality”[tiab] OR “Virtual Medicine”[tiab] OR "virtual care"[tiab] OR computer[ti] OR software[ti] OR "Internet"[ti] OR "web"[ti])

AND

("Diabetes Mellitus, type 2"[Mesh] OR "Prediabetic State"[Mesh] OR “Type 2 Diabetes”[tiab] OR “Diabetes Type 2”[tiab] OR “Type II Diabetes”[tiab] OR “Diabetes Type II”[tiab] OR "Prediabetes"[tiab] OR "Prediabetic"[tiab] OR "Diabetes Prevention Program"[tiab])

AND

("Prevention and Control"[MeSH Subheading] OR "Primary Prevention"[Mesh] OR "prevent*"[tiab] OR "Prophylaxis"[tiab])

AND (2014:2024[pdat])

Embase

('digital health'/exp OR 'telemedicine'/exp OR 'wearable device'/exp OR 'mobile application'/exp OR 'smartphone'/exp OR 'artificial intelligence'/exp OR ‘machine learning’/de OR 'deep learning'/exp OR ‘supervised machine learning’/exp OR ‘support vector machine’/exp OR ‘unsupervised machine learning’/exp OR 'activity tracker'/exp OR 'social media'/exp OR 'video game'/exp OR 'virtual reality'/exp OR 'Internet'/exp OR "digital technology":ti,ab OR "digital tool":ti,ab OR "digital health":ti,ab OR “digital platform*”:ti,ab OR "artificial intelligence":ti,ab OR AI:ti,ab OR “machine learning”:ti,ab OR “federated learning”:ti,ab OR “computational intelligence”:ti,ab OR “machine intelligence”:ti,ab OR "computer reasoning":ti,ab OR “big data”:ti,ab OR "deep learning":ti,ab OR "user-computer interface":ti,ab OR tablet:ti,ab OR iPad:ti,ab OR “Smartphone”:ti,ab OR “Smart phone”:ti,ab OR “Smartwatch”:ti,ab OR "portable game":ti,ab OR "video games":ti,ab OR "digital assistant":ti,ab OR “Digital Therapeutics”:ti,ab OR "social media":ti,ab OR "clinical decision support systems":ti,ab OR "clinical decision support system":ti,ab OR "decision support system":ti,ab OR "decision support systems":ti,ab OR eHealth:ti,ab OR "electronic health":ti,ab OR e-health:ti,ab OR "Telemedicine":ti,ab OR "Tele-medicine":ti,ab OR "Tele medicine":ti,ab OR “mHealth”:ti,ab OR “m-Health”:ti,ab OR “m Health”:ti,ab OR “Mobile Health”:ti,ab OR “MobileHealth”:ti,ab OR “Mobile-Health”:ti,ab OR "mobile technology":ti,ab OR "mobile applications":ti,ab OR "mobile application":ti,ab OR “mobile app”:ti,ab OR "mobile device":ti,ab OR “eHealth”:ti,ab OR “e-Health”:ti,ab OR “e Health”:ti,ab OR “Telehealth”:ti,ab OR “Tele health”:ti,ab OR “Tele-health”:ti,ab OR “Tele monitor*”:ti,ab OR “Tele-monitor*”:ti,ab OR “Telemonitor*”:ti,ab OR “Wearable Electronic Device*”:ti,ab OR “Wearable Device*”:ti,ab OR “Health Tracking Devices”:ti,ab OR “wireless device*”:ti,ab OR “Mobile Phone”:ti,ab OR “MobilePhone”:ti,ab OR “Mobile Telephone”:ti,ab OR “pedometer*”:ti,ab OR “virtual reality”:ti,ab OR “Virtual Medicine”:ti,ab OR "virtual care":ti,ab OR "computer":ti OR "software":ti OR "Internet":ti OR "web":ti)

AND

('non insulin dependent diabetes mellitus'/exp OR 'impaired glucose tolerance'/exp OR “Type 2 Diabetes”:ti,ab OR “Diabetes Type 2”:ti,ab OR “Type II Diabetes”:ti,ab OR “Diabetes Type

II”:ti,ab OR "Prediabetes":ti,ab OR "Prediabetic":ti,ab OR "Diabetes Prevention Program":ti,ab)

AND

('prevention'/exp OR "prevent*":ti,ab OR "Prophylaxis":ti,ab)

AND [2014-2024]/py **AND** ([article]/lim OR [article in press]/lim OR [review]/lim)

CINAHL Complete (EBSCOhost)

(MH "Digital Health+" OR MH "Telemedicine+" OR MH "Wearable Sensors+" OR MH "Mobile Applications" OR MH "Smartphone" OR MH "Artificial Intelligence+" OR MH "Machine Learning+" OR MH "Fitness Trackers" OR MH "Social Media+" OR MH "Video Games+" OR MH "Virtual Reality+" OR MH "Internet+" OR TI("digital technology" OR "digital tool" OR "digital health" OR “digital platform*” OR "artificial intelligence" OR AI OR “machine learning” OR “federated learning” OR “computational intelligence” OR “machine intelligence” OR "computer reasoning" OR “big data” OR "deep learning" OR "user-computer interface" OR tablet OR iPad OR “Smartphone” OR “Smart phone” OR “Smartwatch” OR "portable game" OR "video games" OR "digital assistant" OR “Digital Therapeutics” OR "social media" OR "clinical decision support systems" OR "clinical decision support system" OR "decision support system" OR "decision support systems" OR eHealth OR "electronic health" OR e-health OR "Telemedicine" OR "Tele-medicine" OR "Tele medicine" OR “mHealth” OR “m-Health” OR “m Health” OR “Mobile Health” OR “MobileHealth” OR “Mobile-Health” OR "mobile technology" OR "mobile applications" OR "mobile application" OR “mobile app” OR "mobile device" OR “eHealth” OR “e-Health” OR “e Health” OR “Telehealth” OR “Tele health” OR “Tele-health” OR “Tele monitor*” OR “Tele-monitor*” OR “Telemonitor*” OR “Wearable Electronic Device*” OR “Wearable Device*” OR “Health Tracking Devices” OR “wireless device*” OR “Mobile Phone” OR “MobilePhone” OR “Mobile Telephone” OR “pedometer*” OR “virtual reality” OR “Virtual Medicine” OR "virtual care" OR "computer" OR "software" OR "Internet" OR "web") OR AB("digital technology" OR "digital tool" OR "digital health" OR “digital platform*” OR "artificial intelligence" OR AI OR “machine learning” OR “federated learning” OR “computational intelligence” OR “machine intelligence” OR "computer reasoning" OR “big data” OR "deep learning" OR "user-computer interface" OR tablet OR iPad OR “Smartphone” OR “Smart phone” OR “Smartwatch” OR "portable game" OR "video games" OR "digital assistant" OR “Digital Therapeutics” OR "social media" OR "clinical decision support systems" OR "clinical decision support system" OR "decision support system" OR "decision support systems" OR

eHealth OR "electronic health" OR e-health OR "Telemedicine" OR "Tele-medicine" OR "Tele medicine" OR “mHealth” OR “m-Health” OR “m Health” OR “Mobile Health” OR “MobileHealth” OR “Mobile-Health” OR "mobile technology" OR "mobile applications" OR "mobile application" OR “mobile app” OR "mobile device" OR “eHealth” OR “e-Health” OR “e Health” OR “Telehealth” OR “Tele health” OR “Tele-health” OR “Tele monitor*” OR “Tele-monitor*” OR “Telemonitor*” OR “Wearable Electronic Device*” OR “Wearable Device*” OR “Health Tracking Devices” OR “wireless device*” OR “Mobile Phone” OR “MobilePhone” OR “Mobile Telephone” OR “pedometer*” OR “virtual reality” OR “Virtual Medicine” OR "virtual care"))

AND

(MH "Diabetes Mellitus, Type 2" OR MH "Prediabetic State" OR TI(“Type 2 Diabetes” OR “Diabetes Type 2” OR “Type II Diabetes” OR “Diabetes Type II” OR "Prediabetes" OR "Prediabetic" OR "Diabetes Prevention Program") OR AB(“Type 2 Diabetes” OR “Diabetes Type 2” OR “Type II Diabetes” OR “Diabetes Type II” OR "Prediabetes" OR "Prediabetic" OR "Diabetes Prevention Program"))

AND

(TI("prevent*" OR "Prophylaxis") OR AB("prevent*" OR "Prophylaxis"))

AND (PY 2014-2024)

Web of Science

(TI=("digital technology" OR "digital tool" OR "digital health" OR “digital platform*” OR "artificial intelligence" OR AI OR “machine learning” OR “federated learning” OR “computational intelligence” OR “machine intelligence” OR "computer reasoning" OR “big data” OR "deep learning" OR "user-computer interface" OR tablet OR iPad OR “Smartphone” OR “Smart phone” OR “Smartwatch” OR "portable game" OR "video games" OR "digital assistant" OR “Digital Therapeutics” OR "social media" OR "clinical decision support systems" OR "clinical decision support system" OR "decision support system" OR "decision support systems" OR eHealth OR "electronic health" OR e-health OR "Telemedicine" OR "Tele-medicine" OR "Tele medicine" OR “mHealth” OR “m-Health” OR “m Health” OR “Mobile Health” OR “MobileHealth” OR “Mobile-Health” OR "mobile technology" OR "mobile applications" OR "mobile application" OR “mobile app” OR "mobile device" OR “eHealth” OR “e-Health” OR “e Health” OR “Telehealth” OR “Tele health” OR “Tele-health” OR “Tele monitor*” OR “Tele-monitor*” OR “Telemonitor*” OR “Wearable Electronic Device*” OR “Wearable Device*” OR “Health Tracking Devices” OR “wireless device*” OR

“Mobile Phone” OR “MobilePhone” OR “Mobile Telephone” OR “pedometer*” OR “virtual reality” OR “Virtual Medicine” OR "virtual care" OR "computer" OR "software" OR "Internet" OR "web") OR AB=("digital technology" OR "digital tool" OR "digital health" OR “digital platform*” OR "artificial intelligence" OR AI OR “machine learning” OR “federated learning” OR “computational intelligence” OR “machine intelligence” OR "computer reasoning" OR “big data” OR "deep learning" OR "user-computer interface" OR tablet OR iPad OR “Smartphone” OR “Smart phone” OR “Smartwatch” OR "portable game" OR "video games" OR "digital assistant" OR “Digital Therapeutics” OR "social media" OR "clinical decision support systems" OR "clinical decision support system" OR "decision support system" OR "decision support systems" OR eHealth OR "electronic health" OR e-health OR "Telemedicine" OR "Tele-medicine" OR "Tele medicine" OR “mHealth” OR “m-Health” OR “m Health” OR “Mobile Health” OR “MobileHealth” OR “Mobile-Health” OR "mobile technology" OR "mobile applications" OR "mobile application" OR “mobile app” OR "mobile device" OR “eHealth” OR “e-Health” OR “e Health” OR “Telehealth” OR “Tele health” OR “Tele-health” OR “Tele monitor*” OR “Tele-monitor*” OR “Telemonitor*” OR “Wearable Electronic Device*” OR “Wearable Device*” OR “Health Tracking Devices” OR “wireless device*” OR “Mobile Phone” OR “MobilePhone” OR “Mobile Telephone” OR “pedometer*” OR “virtual reality” OR “Virtual Medicine” OR "virtual care"))

AND

(TI=(“Type 2 Diabetes” OR “Diabetes Type 2” OR “Type II Diabetes” OR “Diabetes Type II” OR "Prediabetes" OR "Prediabetic" OR "Diabetes Prevention Program") OR AB=(“Type 2 Diabetes” OR “Diabetes Type 2” OR “Type II Diabetes” OR “Diabetes Type II” OR "Prediabetes" OR "Prediabetic" OR "Diabetes Prevention Program"))

AND

(TI=("prevent*" OR "Prophylaxis") OR AB=("prevent*" OR "Prophylaxis"))

AND PY=(2014-2024)
