# Supplementary material for: Digital Health Interventions to Prevent Type 2 Diabetes Mellitus: Systematic Review
Source: J Med Internet Res. 2025 Apr 25;27:e67507. doi: 10.2196/67507 (PMC12064978; doi:10.2196/67507)
Supplement: Multimedia Appendix 3 [file jmir_v27i1e67507_app3.pdf]

Multimedia Appendix 3. Data extraction form

Author, Year	Title	Objectives/ Aims	Methods	Country	Study setting	Duration of study	Study design	Baseline Population Characteristics	Explanation of DHI	Digital delivery	Primary users	DHI category	Duration of DHI	HCP interaction	Comparator	Key Results	Retention rate
