# Supplementary material for: Digital Health Interventions to Prevent Type 2 Diabetes Mellitus: Systematic Review
Source: J Med Internet Res. 2025 Apr 25;27:e67507. doi: 10.2196/67507 (PMC12064978; doi:10.2196/67507)
Supplement: Multimedia Appendix 4 [file jmir_v27i1e67507_app4.pdf]

Multimedia Appendix 4. WHO's DHI classification* [1].

Primary user	DHI categories	Explanation
Persons	Targeted communication to persons	Transmit targeted health alerts, reminders, information to persons
	Untargeted communication to persons	Transmit untargeted health alerts, reminders, information to persons
	Person to person communication	Communication between individuals as peers in group or network
	Personal health tracking	Mobile applications, phone based sensors, health records, wearables to monitor individual's health status
	Person based reporting	Enable individuals to report on experiences, issues, with health services
	On demand communication with persons	Platforms allow to access to health (Eg. website, human-like conversation)
	Person-centred financial transactions	To facilitate financial transactions for persons
	Person-centred consent managementd	To manage the provision and withdrawal of consent by individuals about access or share individual's health information to healthcare providers
Healthcare provider	Identification and registration of persons	Verify and enrol individuals into health services
	Person-centred health records	Electronic record (store, access and share health information)
	Healthcare provider decision support	To assist healthcare providers in making clinical decision making
	Telemedicine	To provide healthcare services remotely
	Healthcare provider communication	Communication among healthcare providers
	Referral coordination	To assist in communicating, collaborating in referrals
	Scheduling and activity planning for healthcare providers	To assist in prioritizing, scheduling and planning
	Healthcare provider training	Education and training in digital form
	Prescription and medication management	Track prescription orders, monitor physical consumption
	Laboratory and diagnostics imaging management	To manage, exchange laboratory orders and results
	Healthcare provider financial transactions	To assist financial transactions

*DHI categories with health system managers and data services as primary users are not presented due to their absence in our included studies

Reference

1. World Health Organization. Classification of Digital Interventions, Services and Applications in Health: A Shared Language to Describe the Uses of Digital Technology for Health (2nd Ed.). Geneva: 2023.
