# Supplementary material for: Digital Health Interventions to Prevent Type 2 Diabetes Mellitus: Systematic Review
Source: J Med Internet Res. 2025 Apr 25;27:e67507. doi: 10.2196/67507 (PMC12064978; doi:10.2196/67507)
Supplement: Multimedia Appendix 5 [file jmir_v27i1e67507_app5.pdf]

Multimedia Appendix 5. Study characteristics

Author, Year, Country (Setting)	Design, Duration (I duration)	Sample (Age, BMI, HbA1c as mean (SD)	Comparator	Retention rate (I)	Digital delivery	Primary users (HCP interaction)	DHI category							Quadruple Aims					Bias risk
							TCP	PPC	PHT	DCP	PHR	DS	TM	Population health		Consumer experience	HCP experience	Health care cost	
														Type 2 diabetes development	Dysglycemia				
Aguiar, 2016, Australia (Academic institution) [1]	RCT, 12m (6m)	N=101 (I=53, C=48) Age=52.3 (9.7), Male%=100 BMI=32.4 (3.4) HbA1c=5.8(0.5)	No intervention	89% at 3m 81% at 6m	DVD, Web, Wearables / Trackers	Prs (Non)	✓		✓	✓				NA	Significant HbA1c reduction vs C, P=0.002	NA	NA	NA	High
Alcántara, Aragón, 2018, Spain (Healthcare) [2]	RCT, 12m (12m)	N=183(I=91, C=92) Age=44.5 (10.8) Male%=16.4 BMI (SD)=34.75 (2.75) HbA1c=5.47 (0.36)	Usual care	66% at 6m	Message, Web, Social media, Medical device	Prs (In-Prs, Remote)	✓	✓	✓					NA	No significant differences between groups	NA	NA	NA	Some concerns

Al-Hamdan, 2021, Saudi Arabia (Healthcare) [3]	NRCT, 8m (6m)	N=120 (I1=43, I2=40, C=37) I1: Age=43.7 (8.1), Male%=0 BMI=30 (5.1), FPG 5.7 (1.0) I1=WhatsApp, I2=Group-based	Usual care, Group support	NA	Message, Mobile app, Social media, Smartphone/ Tablet	Prs (In-Prs, Remote)	✓	✓						NA	No significant difference between groups, P=0.33	NA	NA	NA	Low
Arora, 2023, US (Healthcare) [4]	BAS, 22m (12m)	N=163 (I=163 C=0) Age=52.8(12.1) Male%=33.6 BMI=32.8 (6.9) HbA1c=6.0 (0.3)	No comparator	54% at 12m	Message, Web, Wearables / Trackers, Smartphone/ Tablet	Prs (Remot e)	✓		✓					NA	Reduction predicted HbA1c (P<0.001)	NA	NA	NA	Low

Birse, 2022, US (Community) [5]	CS, 39m (24m)	N=2567 (I=379, Non-enrolled group=2188) I: Age: 51(44–57), Male%=27 BMI median=33.3 (29.8–38.3*) HbA1c median=5.6 (5.3–5.8*)	No comparator		Web, Social media, Wearables / Trackers, Medical device, Message, Call	Prs, HCP (Remot e)	✓	✓	✓				✓	NA	Reduction FBG, P<0.001	NA	NA	NA	Low
Block, 2015, US (Academic institution) [6]	RCT, NA (6m)	N=339 (I=163 C=176) Age=55.0 (8.9), Male%=68.7 BMI=31.2 (4.4) HbA1c=5.6 (0.3)	Wait-list control	89.1% at 3m 86.1% at 6m	Message, Interactive voice response, Web, Mobile app, Smartphone/ Tablet	Prs, HCP (In-Prs)	✓		✓	✓			✓	NA	Significantly greater reductions HbA1c, FBG vs C (Ps<0.001)	Minor unrelated study events	NA	NA	Low

Bock, 2019, US (Academic institution) [7]	RCT, 3m (3m)	N=84 (I1=27, I2=28, C=29) Age=51.4 (11.5), Male%=20 HbA1c=5.9 (0.39) I1: Videogames I2: Standard	Usual care, Standard exercise	73.1% sessions at 3m	Games, Call, Medical device	Prs, HCP (In-Prs)	✓		✓				✓	NA	Reduction HbA1c: I1: - 2% vs I2: - 0.6%, C: - 0.6% (Ps≤ 0.03)	Higher motivation vs C, P<0.05	NA	NA	Some concerns
Brazeau, 2014, Canada (Academic institution) [8]	BAS, NA (13m)	N=27 (I=27, C=0) Male%=0, BMI= 29.1 (6.7) FPG=5.8 (0.8)	No comparator	75.0% at 13m	Call, Web, Forum, Wearables/ Trackers	Prs, HCP (Remote)	✓	✓	✓	✓			✓	NA	Reduction FBG, OGTT glucose	NA	NA	NA	Moderate

Castro Sweet, 2018, US (NA) [9]	BAS, 12m (12m)	N=501 (I=501, C=0) Age=68.8 (2.6), Male%=36 BMI=33.6 (5.7)	No comparator		Call, Web, Forum, Mobile app, Wearables/ Trackers, Smartphone/ Tablet, Medical device, Video call	Prs, HCP (In-Prs, Remote)	✓	✓	✓				✓	NA	Reduction HbA1c= -0.14%, P=0.001	NA	NA	NA	Serious
Cha, 2014, US (NA) [10]	BAS, NA (3m)	N=13, Age=24.38 ± 2.24, Male%=23.1 BMI=41.0 (7.3) HbA1c=6.0 (0.5)	No comparator	86.7% at 3m	Email, Call, Web, Mobile app, Smartphone/ Tablet	Prs, HCP (Remot e)	✓		✓	✓			✓	1 developed T2DM, 5 back to euglycemia	Significant reduction HbA1c after 12 weeks, P<0.05	Satisfacti on score = 4.15/5	NA	NA	Moderate

Chung, 2023, Taiwan (Healthcare) [11]	RCT, 15m (3m)	N=121 (I1=42, I2=41, C=38) Age=58.08 (10.21), Male%=47.1 I1: FPG=25.77 (4.47), HbA1c (%): 6.15 (0.32) I1: TCM app, I2: mHealth app	Usual care, mHealth app	NA	Message, Forum, Mobile app, Games, Smartphone/ Tablet	Prs (Remot e)	✓	✓		✓				NA	Reduction HbA1c 3 groups, no significant difference between groups	NA	NA	NA	Low
Dachel, 2021, US (Healthcare) [12]	BAS, 3m (3m)	N=13 (I=13, C=0), Age=60.1, Male%=53.8 BMI=35 (31), HbA1c=5.9	No comparator	100% at 3m	Call, Wearables / Trackers, Smartphone/ Tablet	Prs, HCP (In-Prs, Remote)	✓		✓				✓	NA	No significant reduction HbA1c (P=0.34)	Positive and negative feedback	NA	NA	Serious

Everett, 2018, US (Academic institution, Healthcare) [13]	CS, 13m (3m)	N=38 (I=38, C=0) Age=57.2 (9.1), Male%= 37 BMI median=32.6 (4.5) HbA1c median=6.0 (5.7-6.2*)	No comparator	88.4% at 3m	Message, Mobile app, Wearables/ Trackers, Smartphone/ Tablet, Medical device, AI	Prs (Non)	✓		✓	✓				NA	Median change in HbA1c= -0.1% (P=0.04)	NA	NA	NA	Some concerns
Ferrara, 2016, US (Healthcare) [14]	RCT, 33m (5m)	N=2280 (I=1087, C=1193) Age: 18-39: 92.3%, 40-50: 7.7%, Male%=0 BMI=28.7(6.8), FPG=5.1 (0.7)	Usual care	15.3%	Call	Prs, HCP (Remote)	✓						✓	No significant difference risk prediabetes/T2DM vs C	NA	NA	NA	$78.00 /person, $121.00 (included call costs)	High

Fitzpatrick, 2022, US (Healthcare) [15]	NRCT, 24m (12m)	N=3904 (I=47, C=3432) Age=69.2 (2.9), Male%=44.8 BMI=35.4 (5.1), HbA1c=5.94 (0.18)	Usual care	89% for the full 12m	Email, Message, Call, Web, Social media, Medical device, Wearables/ Trackers	Prs, HCP (Remot e)	✓	✓	✓				✓	Less likely diagnosed T2DM, not statistically significant	Reduction HbA1c vs C at 12m: -0.10% (P<0.05), but not at 24m	NA	NA	NA	Serious
Fukuoka, 2014, US (Healthcare) [16]	RCT, 10m (5m)	N=61 (I=30, C=31) Age=55.2(9.0), Male%=23 HbA1c:<5.7: 38.3%, 5.7-6.4: 61.7%	Usual care	90% at 3m 93% at 5m	Message, Mobile app, Wearables/ Trackers, Smartphone/ Tablet	Prs (In-Prs, Remote)	✓		✓					NA	Not effect HbA1c, FBG	Minor unrelated study events	NA	NA	Low
Holmes, 2018, UK (Healthcare) [17]	RCT, 18m (6m)	N=60 (I=29, C=31) I: Age=34.2 (4.3), Male%=0 BMI=34.1 (6.3) HbA1c=5.4	Usual care	70% at 6m	Message, Call, Wearables/ Trackers	Prs, HCP (In-Prs, Remote)	✓						✓	NA	No significant difference between groups	NA	NA	NA	Low

Karvela, 2024, UK (Healthcare) [18]	RCT, 36m (6.5m)	N=147 (I1=46, I2=50, C=51) Age=60 (50–67), Male%=31 BMI=30.4 (6.9), HbA1c=6.2 I1: Digital group, I2: DNA group	Usual care, Usual care, DNA report	I1: 63.6% I2: 73.0% at 6.5m	Mobile app, Wearables / Trackers , Smartphone/ Tablet	Prs (Non)	✓		✓					Develop T2DM: 6w: I1=2.7 %, I2=4.5 %, C=7.1 % 12w: I1=7.4 %, I2=0%, C=3.6 %	No significant change HbA1c vs C, P=0.31	NA	NA	NA	High
Katula, 2022, US (Healthcare) [19]	RCT, NA (12m)	N=599(I=299, C=300), Age=55.4 (12.7), Male%=38.6 BMI (SD)= 36.0(6.4), HbA1c=5.8 (0.3)	Usual care	>80% at 12m	Message , Web, Forum, Wearables / Trackers , Smartphone/ Tablet	Prs (Remote)	✓	✓	✓					NA	Significantly greater reduction HbA1c vs C (P≤0.001)	Active engagement =17/52 weeks	NA	NA	Some concerns

Khunti, 2021, UK (Healthca re) [20]	RCT, 60m (48m)	N=1366 (I1=456, I2=450, C=460) I1: Age=59.3 (9.1), Male%=50.9 BMI median=28.4, Prediabetes (%): 38.7 I1: Walking away+, I2: Walking away	Leaf let, Leaf let and grou p educ atio n	I1: 68.9% at 48m I2: 66.2% at 48m	Message , Call, Wearables / Trackers	Prs, HC P (Rem ot e)	✓		✓				✓	Odds ratios T2DM at 48m: I1 vs C: 1.25 (97.5% CI 0.57-2.74) I2 vs C: 1.58 (97.5% CI 0.74-3.39)	HbA1c was similar across all three groups	NA	NA	Lifetim e costs= $25,30 5 (standa rd care=$ 24,843 , willing -to- pay thresh old=$2 1,987)	Some concerns
Kim, 2021, South Korea (Healthca re) [21]	NRCT , NA (3m)	N=119 (I=57, C=62) Age=Over 35: 47.9%, Male%=0 I: Weight (kg)=67.15 (12.95) HbA1c=5.56 (0.36)	Writ ten educ atio n	89.1% at 3m	Mobile app, Smartph one/ Tablet, VR	Prs (No n)	✓		✓	✓				NA	Reduction HbA1c, FBG vs C (P=0.031, P=0.019)	NA	NA	NA	Serious

Kitazawa, 2024, Japan (Healthcare) [22]	RCT, 6m (3m)	N=168 (I=82, C=86) I: Age=49.19 (8.75), Male%=80.2 BMI=26.57 (3.77), HbA1c=5.81 (0.34)	No intervention	94.5% at 3m	Message, Mobile app, Smartphone/ Tablet, Medical device	Prs (Non)	✓		✓					NA	Improvement time glucose in range, below range vs C, Ps=0.03	Usability: 11.3 to 6.5 days/fortnight	NA	NA	Low
Lakka, 2023, Finland (Healthcare) [23]	RCT, 22m (12m)	N=2907 (I1=971, I2=967, C=969) I1: Age: 55.2(10.1), Male%=19 BMI =30.9 (5.4), Prediabetes (%): 56 I1: BitHabit+, I2: BitHabit	Usual care, Web app, in-prs group coaching	I1: 83.7% I2: 84.2%	Email, Message, Web, Mobile app, Smartphone/ Tablet	Prs (Non)	✓		✓	✓				NA	Borderline significance HbA1c change (I1 vs C, P=0.05)	NA	NA	NA	Some concerns

Lee, 2021, South Korea (Healthca re) [24]	RCT, NA (6m)	N=415 (I=207, C=208) I: Age=52.6 (7.5), Male%=44.4 BMI=26.5 (3.0), HbA1c=5.78 (0.5)	Usu al care	68.9% at 6m	Message , Call, Web, Wearables / Trackers	Prs, HC P (In-Prs)	✓		✓				✓	NA	No significant reduction glucose vs C (P=0.1)	NA	NA	NA	Some concerns
Lim, 2022, Singapor e (Healthca re) [25]	RCT, 24m (6m)	N=148 (I=72, C=76) I: Age=53.1(9.3), Male%=59.7, BMI=29.8(4.2), HbA1c=5.94 (0.48)	Usu al care	93.1% at 6m	Message , Mobile app, Wearables / Trackers , Medical device, Smartph one/ Tablet	Prs, HC P (Rem ot e)	✓		✓				✓	NA	Reduction HbA1c, FBG vs C (Ps <0.001)	NA	NA	NA	Low
Limaye, 2017, India (Academ ic institutio n) [26]	RCT, 36m (12m)	N=265 (I=133, C=132) I: Age: 36.8(7.2), Male%: 74.4, BMI=27.0(3.2) , FPG=4.12(0.54)	Usu al care	78.9% at 12m	Email, Message , Web, Smartph one/ Tablet, Social media	Prs (Rem ot e)	✓	✓		✓				NA	Reduction FBG vs C (P=0.022)	NA	NA	Increm ental cost=$ 39.36/ partici pant (C: $25.62)	Some concer ns

Mann, 2016, US (Healthcare) [27]	RCT, 24m (6m)	N=54 (I=27, C=27) I: Age=47.5 (11.9), Male%=11.11	Usual care	85.2% at 6m	Wearables / Trackers, Electronic health records	Prs, HCP (In-prs)	✓		✓		✓	✓		NA	No change FBG, HbA1c, P=0.8	NA	NA	NA	Low
McKenzie, 2021, US (NA) [28]	BAS, 24m (24m)	N=96 (I=96, C=0) Age=52(10), Male%=20 BMI=39.2 (7.06)	No comparator	75% at 24m	Web, Forum, Medical device	Prs (Remot e)	✓	✓	✓					No significant difference T2DM prevalence	Significant reduction HbA1c, P<0.001	NA	NA	NA	Moderate
Morales Febles, 2023, Spain (Healthcare) [29]	BAS, 12m (12m)	N=27 (I=27, C=0) Age=54.2(9.6), Male%=67 BMI=30.9 (4.7), HbA1c=5.9 (0.4)	No comparator	81.5 at 12m	Call, Wearables/ Trackers	Prs, HCP (In-Prs, Remote)	✓		✓				✓	NA	HbA1c: Baseline: 5.9 (0.3), 12 m: 6 (0.4)	NA	NA	NA	Serious

Moravcová, 2022, Czech Republic (Healthcare) [30]	RCT, NA (6m)	N=100 (I=50, C=50) Age=43.3 (9.5), Male%=29 BMI=40.1 (6.1) T2DM 10%, prediabetes 23%, insulin resistance 67%	Usual care	92% at 3m 60% at 6m	Message, Call, Forum, Mobile app, Smartphone/ Tablet, Games	Prs, HCP (Remot e)	✓	✓					✓	NA	No significant difference between groups, P=0.6	NA	NA	NA	High
Muralidharan, 2021, India (Community) [31]	RCT, NA (3m)	N=561 (I=271, C=290) I: Age=37.8(9.2), Male%=56.1 BMI=29.4 (3.8) FPG=93.5 (10.6)	Usual care	72% at 6m	Message, Call, Mobile app, Smartphone/ Tablet	Prs, HCP (Remot e)	✓						✓	NA	No significant changes in FBG both group	NA	NA	NA	Low
Nanditha, 2020, India and UK (NA) [32]	RCT, NA (24m)	N=2062 (I=1031, C=1031) Age=52.1 (10.3), Male%=64.1 I: BMI=28.7 (4.7), HbA1c=6.1 (0.1)	Usual care,	90.0% at 24m	Message	Prs (In-Prs)	✓							No significant difference risk T2DM vs C, P=0.22	No significant difference % achieving HbA1c ≤6.0%	Acceptab ility: 50% (India), >82% (UK)	NA	NA	Low

Nicklas, 2014, US (Healthcare) [33]	RCT, NA (6m)	N=75 (I=36, C=39) I: Age=33.6 (4.8), Male%=0 BMI=31.2 (5.8), Prediabetes: 31%	Handout	82% at 6m 88% at 12m	Email, Message, Call, Web, Smartphone/ Tablet, Wearables / Trackers, Medical device	Prs, HCP (Remote)	✓		✓	✓			✓	No significant differences T2DM between groups	No significant differences HbA1c between groups	NA	NA	NA	Some concerns
Patel, 2021, US (Academic institution) [34]	BAS, 10m (6m)	N=186 (I1=93, I2=93) Age= 56.7 (12.7), Male%=33.9 BMI= 32.7 (7.3), HbA1c=6.1 (0.2) I1: Waist-worn group, I2: Wrist- worn group	Wrist-worn, weight scale, message	I1: 81.3% I2: 80.2% (6m)	Message, Wearables / Trackers, Smartphone/ Tablet, Medical device, Mobile app	Prs (Non)	✓		✓					NA	No significant change HbA1c in both groups	NA	NA	NA	Moderate

Peacock, 2015, Australia (Healthcare) [35]	RCT, NA (3m)	N=31 (I=16, C=15)<br>Age=36.0 (4.5),<br>Male%=0<br>BMI=30.3 (8.2)<br>FPG=4.8 (0.8)	No intervention	69% at 3m	Web, Wearables/ Trackers	Prs (In-Prs)	✓		✓					NA	No significant FBG change: 0.3 (0.5), C -0.1 (0.6), P=0.052	Empowered about food choices, P=0.04	NA	NA	Low
Petroni, 2023, Italy (Academic institution) [36]	CS, 60m (1m)	N=546 (I1=212, I2=334)<br>Age=50.6 (11.8),<br>Male%=53.8<br>BMI=33.8 (6.0)<br>HbA1c=6.52(1.4 3)<br>I1=web-based group, I2: group- based group	Group counselling	47.2% at 3m	Web, Games	Prs (Remote)	✓							No significant difference in the time to T2DM between groups	HbA1c increased by 0.18 ± 0.71%	NA	NA	NA	Moderate

Pires, 2022, Bangladesh (Healthcare, Community) [37]	RCT, 24m (14m)	N=1881 (I1=655, I2=594, C=632)<br>I1: Prediabetes%=30.7<br>I1: mHealth group, I2: Participatory group	No intervention, In-prs group meeting	I1: 85%, I2: 84% (14m)	Message	Prs (Non)	✓							No significant impact	No effect on glycemic outcomes	NA	NA	NA	High
Potzel, 2022, Germany (Healthcare, Academic institution) [38]	RCT, 11m (6m)	N=66 (I=33, C=33)<br>I: Age=37.0 (3.1), Male%=0 BMI median=26.0 (22.6–28.9*), Prediabetes 40.9%	Usual care	81% at 6m	Mobile app, Smartphone/ Tablet	Prs, HCP (Remote)	✓			✓			✓	No T2DM difference between visits, P>0.7	No glucose difference between visits, P>0.7	Well accepted, considered helpful by most users	NA	NA	Some concerns
Ranjani, 2020, India (Community) [39]	RCT, 15m (6m)	N=2558 (I=1281, C=1277)<br>Age=31(6.4), Male %=75<br>28% prediabetes, 10% T2DM, 62% normality	T2DM prevention talk	25.2% at 6m	Email, Message, Mobile app, Smartphone/ Tablet	Prs (Non)	✓		✓					NA	Reduction HbA1c: -1.9% vs C, P<0.05	NA	NA	NA	High

Rollo, 2020, Australia (Academic institution) [40]	RCT, NA (6m)	N=42 (I1=15, I2=13, C=14) I1: Age%=34.0 (4.5), Male%=0 BMI=32.8 (4.1) HbA1c=5.1 (0.3) I1: High, I2: Low personalization group	Not change lifestyle, Web platform	I1: 80% at 6m I2: 54% at 6m	Email, Message, Web, Video call	Prs, HCP (Remote)	✓						✓	NA	No significant change HbA1c, P=0.67	NA	NA	NA	Low
Ross, 2022, UK (Healthcare) [41]	CS, NA (12m)	N=3623 (I=3623, C=0) Age=57.8 (12.4) Male%=49, Indeterminate 0.2% BMI=31.1 (6.3), HbA1c=6.1	No comparator	75% at 6m 75% at 12m	Message, Call, Web, Forum, Mobile app, Wearables / Trackers, Smartphone/ Tablet (5 different digital interventions)	Prs, HCP (In-Prs, Remote)	✓	✓	✓				✓	NA	Reduction HbA1c= -1.6 mmol/mol at 12m, P=0.45	NA	NA	NA	Low

Sakane, 2015, Japan (Healthcare) [42]	RCT, NA (12m)	N=2607 (I=1240, C=1367) I: Age=48.9 (7.8), Male%=82.5 BMI=24.4 (3.2), FPG=5.9	Usual care	82.5% at 12m	Email, Call, Wearables / Trackers , Medical device	Prs, HCP (Remote)	✓		✓				✓	Top calls group: significant risk reduction on T2DM vs C, P=0.02	Top calls group: FPG adjusted HR was 0.56 (95% CI 0.34 to 0.94, p=0.028)	Satisfaction score >24/36	NA	NA	High
Salmon, 2022, US (NA) [43]	BAS, NA (6m)	N=1358 (I=1358, C=0) Age=51.6 (11.6), Male%=40.8 64.7% with BMI ≥ 25 FPG: 5.85 (0.3)	No comparator	NA	Call, Web	Prs, HCP (In-Prs, Remote)	✓			✓			✓	NA	Significant reduction FBG: -0.3 (0.6) mmol/L, P<0.001	NA	NA	NA	Low
Savas, 2015, UK (Healthcare) [44]	BAS, NA (6m)	N=55 (I=55, C=0) Age=65.3(12.6), Male%=56.4 BMI=32.02(5.15), FPG= 6.2(0.44)	No comparator		DVD, Call	Prs, HCP (Remote)	✓						✓	12m: 10% back to euglycemia, 5% T2DM	Reduction FBG: -0.11 (P<0.01)	Positive feedback	Strong patient support confid ence	NA	Serious

Sepah, 2015, US (Community) [45]	BAS, NA (24m)	N=220 (I=220, C=0) Age=43.6 (12.4), Male%=17.3 BMI=36.6(7.5) HbA1c= Starters 5.99 (0.07), Completers 6.02 (0.08)	No comparator	59.5% at 24m	Message, Web, Wearables / Trackers, Social media, Medical device	Prs (Remote)	✓	✓	✓					NA	Reductions HbA1c regardless participation level, P<0.001	NA	NA	NA	Serious
Sevilla, Gonzalez, 2022, Mexico (Healthcare) [46]	BAS, NA (3m)	N=77 (I=77, C=0) Age=48.4 (11.3), Male%=30 HbA 1c=5.9 (0.2)	Web, metformin	42% used platform	Web	Prs (In-Prs)	✓							NA	Reduction HbA1c: used platform, -0.07(0.2), not use: 0.01(0.3), P=0.35	Estimated usability = 48%	NA	NA	Moderate
Staite, 2020, UK (Healthcare) [47]	RCT, 12m (12m)	N=200(I=98, C=102) Age=52.28 (7.94), Male%=47 HbA1c=6.0	Wristband, mobile app	70.4 % at 12m	Message, Web, Mobile app, Wearables/ Trackers, Smartphone/ Tablet	Prs (Non)	✓		✓					No significant difference time to T2DM between groups	No treatment effect on HbA1c	NA	NA	NA	High

Summers, 2021, UK (Healthcare) [48]	BAS, NA (12m)	N=45 Prediabetes group: Age=52.42 (13.43), Male%=44 Weight (kg)= 88.72 (6.69) HbA1c= 6.32(0.14)	No comparator	77.8% at 12m	Email, Web, Wearables / Trackers, Smartphone/ Tablet, Social media, AI	Prs (Non)	✓	✓	✓	✓				NA	Reduction HbA1c: -2.35 mmol/mol (P<0.001)	NA	NA	NA	Serious
Tokunaga, Nakawatase, 2014, Japan (Healthcare) [49]	RCT, NA (6m)	N=141(I=70, C=71) I: Age=44.9 (7.6), Male%=67.1, BMI=22.5(2.7) FBG=5.25(0.51)	Usual care	82.5% at 6m 74.6% at 12m	Software	Prs (Remote)	✓							NA	No difference HbA1c, FBG vs C, P=0.73	NA	NA	NA	Some concerns
Toro, Ramos, 2020, US (Healthcare) [50]	RCT, NA (12m)	N=202 (I=103, C=99) I: Age=55.69 (13.63) Male%=26.2, BMI=31.25 (6.43) HbA1c=5.94 (0.18)	Usual care	77.7% at 12m	Message, Web, Mobile app, Wearables / Trackers, Smartphone/ Tablet	Prs (Remote)	✓		✓					NA	Reduction HbA1c= -0.23, C= -0.16, P=0.93	NA	NA	NA	Low

Vahlberg, 2021, Sweden (Healthcare) [51]	RCT, 24m (3m)	N=79 (I=40, C=39) I: Age=63.9 (10), Male%=60.0 BMI=27.3 (4.2) HbA1c=5.6	Usual care	90% at 12m	Message, Wearables / Trackers	Prs (In-Prs)	✓		✓					NA	No significant difference between groups	NA	NA	NA	Some concerns
Vaughan, 2024, US (Healthcare) [52]	NRCT, 15m (12m)	N=58 (I=29, C=29) I: Age=52.3 (10.3), Male%=29.9, Overweight 4.5%, obese 38.8%, HbA1c=5.76 (0.32)	Usual care	85.1% at 12m	Message, Call, Mobile app, Smartphone/ Tablet	Prs, HCP (In-Prs, Remote)	✓						✓	NA	HbA1c: baseline 5.76 (0.32), 15m: 5.77 (0.50)	NA	NA	NA	Serious
Wilson, 2017, US (Community) [53]	NRCT, NA (13m)	N=829 (I=829, C=1268) Age median= 46 (23-68*), Male%=41.6 BMI estimate=33.9(0.3)	No intervention	19.7/52 lessons over 12m	Email, Message, Call, Mobile app, Wearables / Trackers, Social media, Smartphone/ Tablet	Prs, HCP (Remote)	✓	✓	✓				✓	NA	Reduction FBG: -1.49 mg/dL, C: +1.15 mg/dL, P=0.02	NA	NA	NA	Serious

Key: I: Intervention, HCP=Healthcare provider, DHI=Digital health intervention, TCP=Targeted communication to persons, PPC=Person to Person communication, PHT=Personal health

tracking, PHR=Person-centred health records, TM=Telemedicine, DCP=On demand communication with Persons, DS=HCP decision support, RCT=Randomised Controlled Trial, BAS=Before After Study, CS=Cohort Study, NRCT=Non-Randomised Controlled Trials, NA=Not available, C=Control group, w=week, m=month, Prs=Person, HbA1c=Glycated haemoglobin (%), BMI=Body mass index (kg/m2), FPG=Fasting plasma glucose, AI=Artificial intelligent, VR=Virtual reality

*:95% confidence interval reported

References

1. Aguiar EJ, Morgan PJ, Collins CE, Plotnikoff RC, Young MD, Callister R. Efficacy of the Type 2 Diabetes Prevention Using LifeStyle Education Program RCT. American Journal of Preventive Medicine; 2016;50(3):353-64.
2. Alcántara-Aragón V, Rodrigo-Cano S, Lupianez-Barbero A, Martinez MJ, Martinez C, Tapia J, et al. Web Support for Weight-Loss Interventions: PREDIRCAM2 Clinical Trial Baseline Characteristics and Preliminary Results. Diabetes Technology and Therapeutics; 2018;20(5):380-5.
3. Al-Hamdan R, Avery A, Al-Disi D, Sabico S, Al-Daghri NM, McCullough F. Efficacy of lifestyle intervention program for Arab women with prediabetes using social media as an alternative platform of delivery. J Diabetes Investig; 2021;12(10):1872-80.
4. Arora S, Lam CN, Burner E, Menchine M. Implementation and Evaluation of an Automated Text Message-Based Diabetes Prevention Program for Adults With Pre-diabetes. J Diabetes Sci Technol; 2023:19322968231162601.
5. Birse CE, McPhaul MJ, Arellano AR, Fragala MS, Lagier RJ. Impact of a Digital Diabetes Prevention Program on Estimated 8-Year Risk of Diabetes in a Workforce Population. Journal of Occupational and Environmental Medicine; 2022;64(10):881-8.
6. Block G, Azar KM, Romanelli RJ, Block TJ, Hopkins D, Carpenter HA, et al. Diabetes Prevention and Weight Loss with a Fully Automated Behavioral Intervention by Email, Web, and Mobile Phone: A Randomized Controlled Trial Among Persons with Prediabetes. J Med Internet Res; 2015;17(10):e240.
7. Bock BC, Dunsiger SI, Wu WC, Ciccolo JT, Serber ER, Lantini R, et al. Reduction in HbA1c with Exercise videogames among participants with elevated HbA1c: Secondary analysis of the Wii Heart Fitness trial. Diabetes Res Clin Pract; 2019;154:35-42.
8. Brazeau AS, Leong A, Meltzer SJ, Cruz R, DaCosta D, Hendrickson-Nelson M, et al. Group-based activities with on-site childcare and online support improve glucose tolerance in women within 5 years of gestational diabetes pregnancy. Cardiovasc Diabetol; 2014;13:104.
9. Castro Sweet CM, Chiguluri V, Gumpina R, Abbott P, Madero EN, Payne M, et al. Outcomes of a Digital Health Program With Human Coaching for Diabetes Risk Reduction in a Medicare Population. J Aging Health; 2018;30(5):692-710.
10. Cha E, Kim KH, Umpierrez G, Dawkins CR, Bello MK, Lerner HM, et al. A feasibility study to develop a diabetes prevention program for young adults with prediabetes by using digital platforms and a handheld device. Diabetes Educ; 2014;40(5):626-37.
11. Chung HW, Tai CJ, Chang P, Su WL, Chien LY. The Effectiveness of a Traditional Chinese Medicine-Based Mobile Health App for Individuals With Prediabetes: Randomized Controlled Trial. JMIR Mhealth Uhealth; 2023;11:e41099.
12. Dachel TA, Mota D. Technology and Human Connection to Prevent Diabetes in Rural United States. Journal for Nurse Practitioners; 2021;17(9):1137-40 [PMID:153431553. Language: English. Entry Date: 20211123. Revision Date: 20211123.
13. Everett E, Kane B, Yoo A, Dobs A, Mathioudakis N. A Novel Approach for Fully Automated, Personalized Health Coaching for Adults with Prediabetes: Pilot Clinical Trial. J Med Internet Res; 2018;20(2):e72.

14. Ferrara A, Hedderson MM, Brown SD, Albright CL, Ehrlich SF, Tsai AL, et al. The Comparative Effectiveness of Diabetes Prevention Strategies to Reduce Postpartum Weight Retention in Women with Gestational Diabetes Mellitus: The Gestational Diabetes' Effects on Moms (GEM) Cluster Randomized Controlled Trial. Diabetes Care; 2016;39(1):65-74.
15. Fitzpatrick SL, Mayhew M, Rawlings AM, Smith N, Nyongesa DB, Vollmer WM, et al. Evaluating the Implementation of a Digital Diabetes Prevention Program in an Integrated Health Care Delivery System Among Older Adults: Results of a Natural Experiment. Clinical Diabetes; 2022;40(3):345-53 [PMID:158202557. Language: English. Entry Date: 20220816. Revision Date: 20220816.
16. Fukuoka Y, Gay C, Joiner K, Vittinghoff E. A Novel Mobile Phone Delivered Diabetes Prevention Program in Overweight Adults at Risk for Type 2 Diabetes - A Randomized Controlled Trial. Circulation; 2014;130:2.
17. Holmes VA, Draffin CR, Patterson CC, Francis L, Irwin J, McConnell M, et al. Postnatal Lifestyle Intervention for Overweight Women With Previous Gestational Diabetes: A Randomized Controlled Trial. J Clin Endocrinol Metab; 2018;103(7):2478-87.
18. Karvela M, Golden CT, Bell N, Martin-Li S, Bedzo-Nutakor J, Bosnic N, et al. Assessment of the impact of a personalised nutrition intervention in impaired glucose regulation over 26 weeks: a randomised controlled trial. Sci Rep; 2024;14(1):5428.
19. Katula JA, Dressler EV, Kittel CA, Harvin LN, Almeida FA, Wilson KE, et al. Effects of a Digital Diabetes Prevention Program: An RCT. Am J Prev Med; 2022;62(4):567-77.
20. Khunti K, Griffin S, Brennan A, Dallosso H, Davies M, Eborall H, et al. Behavioural interventions to promote physical activity in a multiethnic population at high risk of diabetes: PROPELS three-arm RCT. Health Technol Assess; 2021;25(77):1-190.
21. Kim SH, Kim HJ, Shin G. Self-Management Mobile Virtual Reality Program for Women with Gestational Diabetes. Int J Environ Res Public Health; 2021;18(4).
22. Kitazawa M, Takeda Y, Hatta M, Horikawa C, Sato T, Osawa T, et al. Lifestyle Intervention With Smartphone App and isCGM for People at High Risk of Type 2 Diabetes: Randomized Trial. J Clin Endocrinol Metab; 2024;109(4):1060-70.
23. Lakka TA, Aittola K, Järvelä-Reijonen E, Tilles-Tirkkonen T, Männikkö R, Lintu N, et al. Real-world effectiveness of digital and group-based lifestyle interventions as compared with usual care to reduce type 2 diabetes risk - A stop diabetes pragmatic randomised trial. Lancet Reg Health Eur; 2023;24:100527.
24. Lee JH, Lim SY, Cha SA, Han CJ, Jung AR, Kim KR, et al. Short-Term Effects of the Internet-Based Korea Diabetes Prevention Study: 6-Month Results of a Community-Based Randomized Controlled Trial. Diabetes Metab J; 2021;45(6):960-5.
25. Lim SL, Ong KW, Johal J, Han CY, Yap QV, Chan YH, et al. A Smartphone App-Based Lifestyle Change Program for Prediabetes (D'LITE Study) in a Multiethnic Asian Population: A Randomized Controlled Trial. Frontiers in Nutrition; 2022;8:10.
26. Limaye T, Kumaran K, Joglekar C, Bhat D, Kulkarni R, Nanivadekar A, et al. Efficacy of a virtual assistance-based lifestyle intervention in reducing risk factors for Type 2 diabetes in young employees in the information technology industry in India: LIMIT, a randomized controlled trial. Diabet Med; 2017;34(4):563-8.
27. Mann DM, Palmisano J, Lin JJ. A pilot randomized trial of technology-assisted goal setting to improve physical activity among primary care patients with prediabetes. Prev Med Rep; 2016;4:107-12.
28. McKenzie AL, Athinarayanan SJ, McCue JJ, Adams RN, Keyes M, McCarter JP, et al. Type 2 Diabetes Prevention Focused on Normalization of Glycemia: A Two-Year Pilot Study. Nutrients; 2021;13(3).
29. Morales Febles R, Marrero Miranda D, Jiménez Sosa A, González Rinne A, Cruz Perera C, Rodríguez-Rodríguez AE, et al. Exercise and Prediabetes After Renal Transplantation (EXPRED-I): A Prospective Study. Sports Med Open; 2023;9(1):32.
30. Moravcová K, Karbanová M, Bretschneider MP, Sovová M, Ožana J, Sovová E. Comparing Digital Therapeutic Intervention with an Intensive Obesity Management Program: Randomized Controlled Trial. Nutrients; 2022;14(10).

31. Muralidharan S, Ranjani H, Anjana RM, Gupta Y, Ambekar S, Koppikar V, et al. Change in cardiometabolic risk factors among Asian Indian adults recruited in a mHealth-based diabetes prevention trial. Digit Health; 2021;7:20552076211039032.
32. Nanditha A, Thomson H, Susairaj P, Srivanichakorn W, Oliver N, Godsland IF, et al. A pragmatic and scalable strategy using mobile technology to promote sustained lifestyle changes to prevent type 2 diabetes in India and the UK: a randomised controlled trial. Diabetologia; 2020;63(3):486-96.
33. Nicklas JM, Zera CA, England LJ, Rosner BA, Horton E, Levkoff SE, et al. A web-based lifestyle intervention for women with recent gestational diabetes mellitus: a randomized controlled trial. Obstet Gynecol; 2014;124(3):563-70.
34. Patel MS, Polsky D, Small DS, Park SH, Evans CN, Harrington T, et al. Predicting changes in glycemic control among adults with prediabetes from activity patterns collected by wearable devices. NPJ Digit Med; 2021;4(1):172.
35. Peacock AS, Bogossian FE, Wilkinson SA, Gibbons KS, Kim C, McIntyre HD. A Randomised Controlled Trial to Delay or Prevent Type 2 Diabetes after Gestational Diabetes: Walking for Exercise and Nutrition to Prevent Diabetes for You. Int J Endocrinol; 2015;2015:423717.
36. Petroni ML, Brodosi L, Armandi A, Marchignoli F, Bugianesi E, Marchesini G. Lifestyle Intervention in NAFLD: Long-Term Diabetes Incidence in Subjects Treated by Web- and Group-Based Programs. Nutrients; 2023;15(3).
37. Pires M, Shaha S, King C, Morrison J, Nahar T, Ahmed N, et al. Equity impact of participatory learning and action community mobilisation and mHealth interventions to prevent and control type 2 diabetes and intermediate hyperglycaemia in rural Bangladesh: analysis of a cluster randomised controlled trial. J Epidemiol Community Health; 2022;76(6):586-94.
38. Potzel AL, Gar C, Banning F, Sacco V, Fritsche A, Fritsche L, et al. A novel smartphone app to change risk behaviors of women after gestational diabetes: A randomized controlled trial. PLoS One; 2022;17(4):e0267258.
39. Ranjani H, Nitika S, Anjana R, Ramalingam S, Mohan V, Saligram N. Impact of Noncommunicable Disease Text Messages Delivered Via an App in Preventing and Managing Lifestyle Diseases: Results of the "myArogya" Worksite-Based Effectiveness Study From India. Journal of Diabetology; 2020;11(2):90-+.
40. Rollo ME, Baldwin JN, Hutchesson M, Aguiar EJ, Wynne K, Young A, et al. The Feasibility and Preliminary Efficacy of an eHealth Lifestyle Program in Women with Recent Gestational Diabetes Mellitus: A Pilot Study. Int J Environ Res Public Health; 2020;17(19).
41. Ross JAD, Barron E, McGough B, Valabhji J, Daff K, Irwin J, et al. Uptake and impact of the English National Health Service digital diabetes prevention programme: observational study. BMJ Open Diabetes Res Care; 2022;10(3).
42. Sakane N, Kotani K, Takahashi K, Sano Y, Tsuzaki K, Okazaki K, et al. Effects of telephone-delivered lifestyle support on the development of diabetes in participants at high risk of type 2 diabetes: J-DOIT1, a pragmatic cluster randomised trial. BMJ Open; 2015;5(8):e007316.
43. Salmon MK, Gordon NF, Constantinou D, Reid KS, Wright BS, Kridl TL, et al. Comparative Effectiveness of Lifestyle Intervention on Fasting Plasma Glucose in Normal Weight Versus Overweight and Obese Adults With Prediabetes. Am J Lifestyle Med; 2022;16(3):334-41.
44. Savas LA, Grady K, Cotterill S, Summers L, Boaden R, Gibson JM. Prioritising prevention: Implementation of IGT Care Call, a telephone based service for people at risk of developing type 2 diabetes. Primary Care Diabetes; 2015;9(1):3-8.
45. Sepah SC, Jiang L, Peters AL. Long-term outcomes of a Web-based diabetes prevention program: 2-year results of a single-arm longitudinal study. J Med Internet Res; 2015;17(4):e92.
46. Sevilla-Gonzalez MDR, Bourguet-Ramirez B, Lazaro-Carrera LS, Martagon-Rosado AJ, Gomez-Velasco DV, Viveros-Ruiz TL. Evaluation of a Web Platform to Record Lifestyle Habits in Subjects at Risk of Developing Type 2 Diabetes in a Middle-Income Population: Prospective Interventional Study. JMIR Diabetes; 2022;7(1):e25105.
47. Staite E, Bayley A, Al-Ozairi E, Stewart K, Hopkins D, Rundle J, et al. A Wearable Technology Delivering a Web-Based Diabetes Prevention Program to People at High Risk of Type 2 Diabetes: Randomized Controlled Trial. JMIR Mhealth Uhealth; 2020;8(7):e15448.

48. Summers C, Tobin S, Unwin D. Evaluation of the Low Carb Program Digital Intervention for the Self-Management of Type 2 Diabetes and Prediabetes in an NHS England General Practice: Single-Arm Prospective Study. JMIR Diabetes; 2021;6(3):e25751.
49. Tokunaga-Nakawatase Y, Nishigaki M, Taru C, Miyawaki I, Nishida J, Kosaka S, et al. Computer-supported indirect-form lifestyle-modification support program using Lifestyle Intervention Support Software for Diabetes Prevention (LISS-DP) for people with a family history of type 2 diabetes in a medical checkup setting: A randomized controlled trial. Prim Care Diabetes; 2014;8(3):207-14.
50. Toro-Ramos T, Michaelides A, Anton M, Karim Z, Kang-Oh L, Argyrou C, et al. Mobile Delivery of the Diabetes Prevention Program in People With Prediabetes: Randomized Controlled Trial. JMIR Mhealth Uhealth; 2020;8(7):e17842.
51. Vahlberg BM, Lundström E, Eriksson S, Holmback U, Cederholm T. Potential effects on cardiometabolic risk factors and body composition by short message service (SMS)-guided training after recent minor stroke or transient ischaemic attack: Post hoc analyses of the STROKEWALK randomised controlled trial. BMJ Open; 2021;11(10).
52. Vaughan EM, Cardenas VJ, Chan W, Amspoker AB, Johnston CA, Virani SS, et al. Implementation and Evaluation of a mHealth-Based Community Health Worker Feedback Loop for Hispanics with and at Risk for Diabetes. Journal of General Internal Medicine; 2024;39(2):229-38.
53. Wilson MG, Castro Sweet CM, Edge MD, Madero EN, McGuire M, Pilsmaker M, et al. Evaluation of a Digital Behavioral Counseling Program for Reducing Risk Factors for Chronic Disease in a Workforce. J Occup Environ Med; 2017;59(8):e150-e5.
