# Supplementary material for: Digital Health Interventions to Prevent Type 2 Diabetes Mellitus: Systematic Review
Source: J Med Internet Res. 2025 Apr 25;27:e67507. doi: 10.2196/67507 (PMC12064978; doi:10.2196/67507)
Supplement: Multimedia Appendix 7 [file jmir_v27i1e67507_app7.pdf]

Multimedia Appendix 7. Effects of DHIs on the Quadruple Aims.

Table S3. Outcome assessment for each study

Study ID	DHI category							DHI subcategory												Effects on Quadruple Aims				
																				Population health		Consumer experience	HCP experience	Health care cost
	TCP	PPC	PHT	DCP	PHR	DS	TM	TCP1	TCP2	PPC1	PHT1	PHT2	DCP1	PCP2	PHR1	PHR2	DS1	TM1	TM2	T2DM development	Disglycemia			
Aguiar, 2016, [1]	RCT, 12m (6m)		✓	✓				✓	✓		✓		✓							NA	+	NA	NA	NA
Alcántara, Aragón, 2018, [2]	RCT, 12m (12m)	✓	✓					✓	✓	✓	✓	✓								NA	0	NA	NA	NA

Al-Hamdan, 2021, [3]	NRCT, 8m (6m)	✓						✓	✓	✓										NA	0	NA	NA	NA
Arora, 2023 [4]	BAS, 22m (12m)		✓					✓	✓		✓									NA	+	NA	NA	NA
Birse, 2022, [5]	CS, 39m (24m)	✓	✓				✓	✓	✓	✓		✓						✓		NA	+	NA	NA	NA

Block, 2015, [6]	RCT, NA (6m)		✓	✓			✓	✓	✓			✓	✓					✓		NA	+	0	NA	NA
Bock, 2019 [7]	RCT, 3m (3m)		✓				✓	✓	✓		✓							✓		NA	+	+	NA	NA
Brazeau, 2014, [8]	BAS, NA (13m)	✓	✓	✓			✓	✓	✓	✓	✓		✓					✓		NA	NA	NA	NA	NA

Castro Sweet, 2018 [9]	BAS, 12m (12m)	✓	✓				✓	✓	✓	✓	✓	✓						✓	✓	NA	+	NA	NA	NA
Cha, 2014 [10]	BAS, NA (3m)		✓	✓			✓	✓	✓									✓	✓	NA	+	+	NA	NA
Chung, 2023, [11]	RCT, 15m (3m)	✓		✓				✓	✓	✓			✓							NA	0	NA	NA	NA

Dachel, 2021, [12]	BAS, 3m (3m)		✓				✓	✓	✓		✓							✓		NA	0	+/-	NA	NA
Everett, 2018, [13]	CS, 13m (3m)		✓	✓				✓	✓		✓	✓	✓	✓						NA	+	NA	NA	NA
Ferrara, 2016, [14]	RCT, 33m (5m)						✓	✓	✓									✓		0	NA	NA	NA	0

Fitzpatrick, 2022, [15]	NRCT, 24m (12m)	✓	✓				✓	✓	✓	✓	✓	✓						✓		0	+	NA	NA	NA
Fukuoka, 2014, [16]	RCT, 10m (5m)		✓					✓	✓		✓									NA	0	0	NA	NA
Holmes, 2018, [17]	RCT, 18m (6m)						✓	✓	✓									✓		NA	0	NA	NA	NA

Karvela, 2024, [18]	RCT, 36m (6.5m)		✓					✓	✓		✓									NA	0	NA	NA	NA
Katula, 2022, [19]	RCT, NA (12m)	✓	✓					✓	✓	✓	✓	✓								NA	+	-	NA	NA
Khunti, 2021, [20]	RCT, 60m (48m)		✓				✓	✓	✓		✓							✓		NA	NA	NA	NA	-

Kim, 2021 [21]	NRCT, NA (3m)		✓	✓				✓	✓			✓		✓						NA	+	NA	NA	NA
Kitazawa, 2024, [22]	RCT, 6m (3m)		✓					✓	✓		✓	✓								NA	+	-	NA	NA
Lakka, 2023 [23]	RCT, 22m (12m)		✓	✓				✓	✓			✓	✓							NA	+	NA	NA	NA

Lee, 2021, [24]	RCT, NA (6m)		✓				✓	✓	✓		✓	✓						✓	✓	NA	0	NA	NA	NA
Lim, 2022, [25]	RCT, 24m (6m)		✓				✓	✓	✓									✓	✓	NA	+	NA	NA	NA
Limaye, 2017 [26]	RCT, 36m(12m)	✓		✓				✓	✓	✓			✓							NA	+	NA	NA	-

Mann, 2016, [27]	RCT, 24m (6m)		✓		✓	✓		✓	✓		✓				✓	✓	✓			NA	0	NA	NA	NA
McKenzie, 2021 [28]	BAS, 24m (24m)	✓	✓					✓	✓	✓	✓									0	+	NA	NA	NA
Morales Febles, 2023, [29]	BAS, 12m (12m)		✓				✓	✓	✓		✓							✓	✓	NA	NA	NA	NA	NA

Moravcová, 2022, [30]	RCT, NA (6m)	✓					✓	✓	✓	✓								✓		NA	0	NA	NA	NA
Muralidharan, 2021, [31]	RCT, NA (3m)						✓	✓	✓									✓		NA	0	NA	NA	NA
Nanditha, 2020, [32]	RCT, NA (24m)							✓	✓											0	0	+	NA	NA

Nicklas, 2014, [33]	RCT, NA (6m)		✓	✓			✓	✓	✓		✓	✓	✓					✓	✓	0	0	NA	NA	NA
Patel, 2021, [34]	BAS, 10m (6m)		✓					✓	✓		✓									NA	0	NA	NA	NA
Peacock, 2015, [35]	RCT, NA (3m)		✓					✓	✓		✓									NA	0	+	NA	NA

Petroni, 2023, [36]	CS, 60m (1m)							✓	✓											0	NA	NA	NA	NA
Pires, 2022 [37]	✓							✓	✓											0	0	NA	NA	NA
Potzel, 2022, [38]	✓			✓			✓	✓	✓				✓					✓		0	0	+	NA	NA

Ranjani, 2020, [39]	✓		✓					✓	✓			✓								NA	+	NA	NA	NA
Rollo, 2020 [40]	✓						✓	✓	✓									✓		NA	0	NA	NA	NA
Ross, 2022 , [41]	✓	✓	✓				✓	✓	✓	✓	✓							✓		NA	0	NA	NA	NA
Sakane, 2015, [42]	✓		✓				✓	✓	✓									✓	✓	+	+	+	NA	NA

Salmon, 2022 [43]	✓			✓			✓	✓	✓				✓					✓		NA	+	NA	NA	NA
Savas, 2015, [44]	✓						✓	✓	✓									✓		NA	+	+	+	NA
Sepah, 2015, [45]	✓	✓	✓					✓	✓	✓	✓									NA	+	NA	NA	NA
Sevilla, Gonzalez , 2022 [46]	✓							✓	✓											NA	0	-	NA	NA

Staite, 2020, [47]	✓		✓					✓	✓		✓									0	0	NA	NA	NA
Summers, 2021, [48]	✓	✓	✓	✓				✓	✓	✓	✓	✓	✓	✓						NA	+	NA	NA	NA
Tokunaga, Nakawatase, 2014, [49]	✓							✓	✓											NA	0	NA	NA	NA
Toro, Ramos, 2020, [50]	✓		✓					✓	✓			✓								NA	0	NA	NA	NA

Vahlberg, 2021 [51]	✓		✓					✓	✓		✓									NA	0	NA	NA	NA
Vaughan, 2024, [52]	✓						✓	✓	✓									✓		NA	NA	NA	NA	NA
Wilson, 2017, [53]	✓	✓	✓				✓	✓	✓	✓	✓							✓		NA	+	NA	NA	NA

Key: DHI=Digital health intervention, TCP=Targeted communication to persons, PPC=Person to Person communication, PHT=Personal health tracking, PHR=Person-centred health records, DCP=On demand communication with Persons, DS=HCP decision support, TM=Telemedicine

Table S4. Outcome assessment for each DHI on T2DM development

DHI category		+	-	0	+/-	Total studies, n (%)
TCP	TCP1	[42]	NA	[33],[15],[38],[14],[37],[32],[47],[28],[36]	NA	10 (18.9%)
	TCP2	[42]	NA	[33],[15],[38],[14],[37],[32],[47],[28],[36]	NA	10 (18.9%)
PPC	PPC1	NA	NA	[15],[28]	NA	2 (3.8%)
PHT	PHT1	NA	NA	[33],[15],[47] [28]	NA	4 (7.5%)
	PHT2	NA	NA	[33],[15]	NA	2 (3.8%)
DCP	DCP1	NA	NA	[33],[38]	NA	2 (3.8%)
	DCP2	NA	NA	NA	NA	0 (0.0%)
PHR	PHR1	NA	NA	NA	NA	0 (0.0%)
	PHR2	NA	NA	NA	NA	0 (0.0%)
DS	DS1	NA	NA	NA	NA	0 (0.0%)
TM	TM1	[42]	NA	[33],[15],[38],[14]	NA	5 (9.4%)
	TM2	[42]	NA	[33]	NA	2 (3.8%)

Key: DHI=Digital health intervention, TCP=Targeted communication to persons, PPC=Person to Person communication, PHT=Personal health tracking, PHR=Person-centred health records, DCP=On demand communication with Persons, DS=HCP decision support, TM=Telemedicine

TCP1=Transmit targeted health information to persons, TCP2=Transmit targeted alerts and reminders to persons, PPC1=Peer group for individuals, PHT1=Self-monitoring of health, PHT2=Active data capture/documentation, DCP1=Look-up of information on health, DCP2=Simulated human-like conversations, PHR1=Manage person-centred structured clinical records, PHR2=Manage person-centred

unstructured clinical records, DS1=Provide prompts and alerts based according to protocol, TM1=Consultations between remote person and healthcare provider, TM2=Remote monitoring of person's health or diagnostic data by provider, NA=Not available

Table S5. Outcome assessment for each DHI on Dysglycaemia

DHI category		+	-	0	+/-	Studies, n (%)
TCP	TCP1	[7],[1],[42],[10],[53],[15],[23],[26],[48],[39],[21],[25],[44],[43],[9],[6],[13],[22],[45],[4],[28],[5],[19]	NA	[51],[33],[38],[18],[12],[37],[32],[11],[30],[3],[16],[50],[31],[17],[41],[47],[2],[40],[49],[27],[34],[24],[46],[35]	NA	47 (88.7%)
	TCP2	[7],[1],[42],[10],[53],[15],[23],[26],[48],[39],[21],[25],[44],[43],[9],[6],[13],[22],[45],[4],[28],[5],[19]	NA	[51],[33],[38],[18],[12],[37],[32],[11],[30],[3],[16],[50],[31],[17],[41],[47],[2],[40],[49],[27],[34],[24],[46],[35]	NA	47 (88.7%)
PPC	PPC1	[53],[15],[26],[48],[9],[45],[19],[28],[5]	NA	[11],[30],[3],[41],[2]	NA	14 (26.4%)
PHT	PHT1	[7],[1],[53],[15],[48],[9],[22],[13],[45],[19],[4],[28]	NA	[51],[33],[18],[12],[16],[41],[47],[2],[27],[34],[24],[35]	NA	24 (45.3%)
	PHT2	[15],[23],[48],[39],[21],[9],[6],[22],[13],[19],[5]	NA	[33],[50],[2],[24]	NA	15 (28.3%)
DCP	DCP1	[1],[23],[26],[48],[43],[6],[13]	NA	[33],[38],[11]	NA	10 (18.9%)

	DCP2	[48],[21],[13]	NA	NA	NA	3(5.7%)
PHR	PHR1	NA	NA	[27]	NA	1(1.9%)
	PHR2	NA	NA	[27]	NA	1(1.9%)
DS	DS1	NA	NA	[27]	NA	1(1.9%)
TM	TM1	[7],[42],[10],[53],[15],[25],[44],[43],[9],[6],[5]	NA	[33],[38],[12],[30],[31],[17],[41],[40],[24]	NA	20(37.7%)
	TM2	NA	NA	[33],[24]	NA	2(3.8%)

Key: DHI=Digital health intervention, TCP=Targeted communication to persons, PPC=Person to Person communication, PHT=Personal health tracking, PHR=Person-centred health records, DCP=On demand communication with Persons, DS=HCP decision support, TM=Telemedicine

TCP1=Transmit targeted health information to persons, TCP2=Transmit targeted alerts and reminders to persons, PPC1=Peer group for individuals, PHT1=Self-monitoring of health, PHT2=Active data capture/documentation, DCP1=Look-up of information on health, DCP2=Simulated human-like conversations, PHR1=Manage person-centred structured clinical records, PHR2=Manage person-centred unstructured clinical records, DS1=Provide prompts and alerts based according to protocol, TM1=Consultations between remote person and healthcare provider, TM2=Remote monitoring of person’s health or diagnostic data by provider, NA=Not available

Table S6. Outcome assessment for each DHI on consumer experience

DHI category		+	-	0	+/-	Studies, n (%)
TCP	TCP1	[7],[42],[10],[38],[44],[32],[35]	[22],[19],[46]	[6],[16]	[12]	13(24.5%)
	TCP2	[7],[42],[10],[38],[44],[32],[35]	[22],[19],[46]	[6],[16]	[12]	13(24.5%)
PPC	PPC1	NA	[19]	NA	NA	1(1.9%)
PHT	PHT1	[7],[35]	[22],[19]	[16]	[12]	6(11.3%)
	PHT2	NA	[22],[19]	[6]	NA	3(5.7%)
DCP	DCP1	[38]	NA	[6]	NA	2(3.8%)
	DCP2	NA	NA	NA	NA	0 (0.0%)
PHR	PHR1	NA	NA	NA	NA	0 (0.0%)
	PHR2	NA	NA	NA	NA	0 (0.0%)
DS	DS1	NA	NA	NA	NA	0 (0.0%)
TM	TM1	[7],[42],[10],[38],[44]	NA	[6]	[12]	7(13.2%)
	TM2	[42],[10]	NA	NA	NA	2(3.8%)

Key: DHI=Digital health intervention, TCP=Targeted communication to persons, PPC=Person to Person communication, PHT=Personal health tracking, PHR=Person-centred health records, DCP=On demand communication with Persons, DS=HCP decision support, TM=Telemedicine

TCP1=Transmit targeted health information to persons, TCP2=Transmit targeted alerts and reminders to persons, PPC1=Peer group for individuals, PHT1=Self-monitoring of health, PHT2=Active data capture/documentation, DCP1=Look-up of information on health, DCP2=Simulated human-like conversations, PHR1=Manage person-centred structured clinical records, PHR2=Manage person-centred

unstructured clinical records, DS1=Provide prompts and alerts based according to protocol, TM1=Consultations between remote person and healthcare provider, TM2=Remote monitoring of person's health or diagnostic data by provider, NA=Not available

Table S7. Outcome assessment for each DHI on HCP experience

**DHI category**		+	-	**0**	+/-	Studies, n (%)
TCP	TCP1	[44]	NA	NA	NA	1(1.9%)
	TCP2	[44]	NA	NA	NA	1(1.9%)
PPC	PPC1	NA	NA	NA	NA	0 (0.0%)
PHT	PHT1	NA	NA	NA	NA	0 (0.0%)
	PHT2	NA	NA	NA	NA	0 (0.0%)
DCP	DCP1	NA	NA	NA	NA	0 (0.0%)
	DCP2	NA	NA	NA	NA	0 (0.0%)
PHR	PHR1	NA	NA	NA	NA	0 (0.0%)
	PHR2	NA	NA	NA	NA	0 (0.0%)
DS	DS1	NA	NA	NA	NA	0 (0.0%)
TM	TM1	[44]	NA	NA	NA	1(1.9%)
	TM2	NA	NA	NA	NA	0 (0.0%)

Key: DHI=Digital health intervention, TCP=Targeted communication to persons, PPC=Person to Person communication, PHT=Personal health tracking, PHR=Person-centred health records, DCP=On demand communication with Persons, DS=HCP decision support, TM=Telemedicine

TCP1=Transmit targeted health information to persons, TCP2=Transmit targeted alerts and reminders to persons, PPC1=Peer group for individuals, PHT1=Self-monitoring of health, PHT2=Active data capture/documentation, DCP1=Look-up of information on health, DCP2=Simulated human-like conversations, PHR1=Manage person-centred structured clinical records, PHR2=Manage person-centred unstructured clinical records, DS1=Provide prompts and alerts based according to protocol, TM1=Consultations between remote person

and healthcare provider, TM2=Remote monitoring of person's health or diagnostic data by provider, HCP experience=Healthcare provider experience, NA=Not available

Table S8. Outcome assessment for each DHI on healthcare cost

DHI category		+	-	0	+/-	Studies, n (%)
TCP	TCP1	NA	[26],[20]	[14]	NA	3(5.7%)
	TCP2	NA	[26],[20]	[14]	NA	2(3.8%)
PPC	PPC1	NA	[26]	NA	NA	1(1.9%)
PHT	PHT1	NA	[20]	NA	NA	1(1.9%)
	PHT2	NA	NA	NA	NA	0 (0.0%)
DCP	DCP1	NA	[26]	NA	NA	1(1.9%)
	DCP2	NA	NA	NA	NA	0 (0.0%)
PHR	PHR1	NA	NA	NA	NA	0 (0.0%)
	PHR2	NA	NA	NA	NA	0 (0.0%)
DS	DS1	NA	NA	NA	NA	0 (0.0%)
TM	TM1	NA	[20]	[14]	NA	2(3.8%)
	TM2	NA	NA	NA	NA	0 (0.0%)

Key: DHI=Digital health intervention, TCP=Targeted communication to persons, PPC=Person to Person communication, PHT=Personal health tracking, PHR=Person-centred health records, DCP=On demand communication with Persons, DS=HCP decision support, TM=Telemedicine

TCP1=Transmit targeted health information to persons, TCP2=Transmit targeted alerts and reminders to persons, PPC1=Peer group for individuals, PHT1=Self-monitoring of health, PHT2=Active data capture/documentation, DCP1=Look-up of information on health, DCP2=Simulated human-like conversations, PHR1=Manage person-centred structured clinical records, PHR2=Manage person-centred

unstructured clinical records, DS1=Provide prompts and alerts based according to protocol, TM1=Consultations between remote person and healthcare provider, TM2=Remote monitoring of person's health or diagnostic data by provider, NA=Not available

References

1. Aguiar EJ, Morgan PJ, Collins CE, Plotnikoff RC, Young MD, Callister R. Efficacy of the Type 2 Diabetes Prevention Using Life Style Education Program RCT. American Journal of Preventive Medicine; 2016;50(3):353-64.
2. Alcántara-Aragón V, Rodrigo-Cano S, Lupianez-Barbero A, Martinez MJ, Martinez C, Tapia J, et al. Web Support for Weight-Loss Interventions: PREDIRCAM2 Clinical Trial Baseline Characteristics and Preliminary Results. Diabetes Technology and Therapeutics; 2018;20(5):380-5.
3. Al-Hamdan R, Avery A, Al-Disi D, Sabico S, Al-Daghri NM, McCullough F. Efficacy of lifestyle intervention program for Arab women with prediabetes using social media as an alternative platform of delivery. J Diabetes Investig; 2021;12(10):1872-80.
4. Arora S, Lam CN, Burner E, Menchine M. Implementation and Evaluation of an Automated Text Message-Based Diabetes Prevention Program for Adults With Pre-diabetes. J Diabetes Sci Technol; 2023:19322968231162601.
5. Birse CE, McPhaul MJ, Arellano AR, Fragala MS, Lagier RJ. Impact of a Digital Diabetes Prevention Program on Estimated 8-Year Risk of Diabetes in a Workforce Population. Journal of Occupational and Environmental Medicine; 2022;64(10):881-8.
6. Block G, Azar KM, Romanelli RJ, Block TJ, Hopkins D, Carpenter HA, et al. Diabetes Prevention and Weight Loss with a Fully Automated Behavioral Intervention by Email, Web, and Mobile Phone: A Randomized Controlled Trial Among Persons with Prediabetes. J Med Internet Res; 2015;17(10):e240.
7. Bock BC, Dunsiger SI, Wu WC, Ciccolo JT, Serber ER, Lantini R, et al. Reduction in HbA1c with Exercise videogames among participants with elevated HbA1c: Secondary analysis of the Wii Heart Fitness trial. Diabetes Res Clin Pract; 2019;154:35-42.
8. Brazeau AS, Leong A, Meltzer SJ, Cruz R, DaCosta D, Hendrickson-Nelson M, et al. Group-based activities with on-site childcare and online support improve glucose tolerance in women within 5 years of gestational diabetes pregnancy. Cardiovasc Diabetol; 2014;13:104.
9. Castro Sweet CM, Chiguluri V, Gumpina R, Abbott P, Madero EN, Payne M, et al. Outcomes of a Digital Health Program With Human Coaching for Diabetes Risk Reduction in a Medicare Population. J Aging Health; 2018;30(5):692-710.
10. Cha E, Kim KH, Umpierrez G, Dawkins CR, Bello MK, Lerner HM, et al. A feasibility study to develop a diabetes prevention program for young adults with prediabetes by using digital platforms and a handheld device. Diabetes Educ; 2014;40(5):626-37.
11. Chung HW, Tai CJ, Chang P, Su WL, Chien LY. The Effectiveness of a Traditional Chinese Medicine-Based Mobile Health App for Individuals With Prediabetes: Randomized Controlled Trial. JMIR Mhealth Uhealth; 2023;11:e41099.
12. Dachel TA, Mota D. Technology and Human Connection to Prevent Diabetes in Rural United States. Journal for Nurse Practitioners; 2021;17(9):1137-40 [PMID:153431553. Language: English. Entry Date: 20211123. Revision Date: 20211123.
13. Everett E, Kane B, Yoo A, Dobs A, Mathioudakis N. A Novel Approach for Fully Automated, Personalized Health Coaching for Adults with Prediabetes: Pilot Clinical Trial. J Med Internet Res; 2018;20(2):e72.

14. Ferrara A, Hedderson MM, Brown SD, Albright CL, Ehrlich SF, Tsai AL, et al. The Comparative Effectiveness of Diabetes Prevention Strategies to Reduce Postpartum Weight Retention in Women with Gestational Diabetes Mellitus: The Gestational Diabetes' Effects on Moms (GEM) Cluster Randomized Controlled Trial. Diabetes Care; 2016;39(1):65-74.
15. Fitzpatrick SL, Mayhew M, Rawlings AM, Smith N, Nyongesa DB, Vollmer WM, et al. Evaluating the Implementation of a Digital Diabetes Prevention Program in an Integrated Health Care Delivery System Among Older Adults: Results of a Natural Experiment. Clinical Diabetes; 2022;40(3):345-53 [PMID:158202557. Language: English. Entry Date: 20220816. Revision Date: 20220816.
16. Fukuoka Y, Gay C, Joiner K, Vittinghoff E. A Novel Mobile Phone Delivered Diabetes Prevention Program in Overweight Adults at Risk for Type 2 Diabetes - A Randomized Controlled Trial. Circulation; 2014;130:2.
17. Holmes VA, Draffin CR, Patterson CC, Francis L, Irwin J, McConnell M, et al. Postnatal Lifestyle Intervention for Overweight Women With Previous Gestational Diabetes: A Randomized Controlled Trial. J Clin Endocrinol Metab; 2018;103(7):2478-87.
18. Karvela M, Golden CT, Bell N, Martin-Li S, Bedzo-Nutakor J, Bosnic N, et al. Assessment of the impact of a personalised nutrition intervention in impaired glucose regulation over 26 weeks: a randomised controlled trial. Sci Rep; 2024;14(1):5428.
19. Katula JA, Dressler EV, Kittel CA, Harvin LN, Almeida FA, Wilson KE, et al. Effects of a Digital Diabetes Prevention Program: An RCT. Am J Prev Med; 2022;62(4):567-77.
20. Khunti K, Griffin S, Brennan A, Dallosso H, Davies M, Eborall H, et al. Behavioural interventions to promote physical activity in a multiethnic population at high risk of diabetes: PROPELS three-arm RCT. Health Technol Assess; 2021;25(77):1-190.
21. Kim SH, Kim HJ, Shin G. Self-Management Mobile Virtual Reality Program for Women with Gestational Diabetes. Int J Environ Res Public Health; 2021;18(4).
22. Kitazawa M, Takeda Y, Hatta M, Horikawa C, Sato T, Osawa T, et al. Lifestyle Intervention With Smartphone App and isCGM for People at High Risk of Type 2 Diabetes: Randomized Trial. J Clin Endocrinol Metab; 2024;109(4):1060-70.
23. Lakka TA, Aittola K, Järvelä-Reijonen E, Tilles-Tirkkonen T, Männikkö R, Lintu N, et al. Real-world effectiveness of digital and group-based lifestyle interventions as compared with usual care to reduce type 2 diabetes risk - A stop diabetes pragmatic randomised trial. Lancet Reg Health Eur; 2023;24:100527.
24. Lee JH, Lim SY, Cha SA, Han CJ, Jung AR, Kim KR, et al. Short-Term Effects of the Internet-Based Korea Diabetes Prevention Study: 6-Month Results of a Community-Based Randomized Controlled Trial. Diabetes Metab J; 2021;45(6):960-5.
25. Lim SL, Ong KW, Johal J, Han CY, Yap QV, Chan YH, et al. A Smartphone App-Based Lifestyle Change Program for Prediabetes (D'LITE Study) in a Multiethnic Asian Population: A Randomized Controlled Trial. Frontiers in Nutrition; 2022;8:10.
26. Limaye T, Kumaran K, Joglekar C, Bhat D, Kulkarni R, Nanivadekar A, et al. Efficacy of a virtual assistance-based lifestyle intervention in reducing risk factors for Type 2 diabetes in young employees in the information technology industry in India: LIMIT, a randomized controlled trial. Diabet Med; 2017;34(4):563-8.
27. Mann DM, Palmisano J, Lin JJ. A pilot randomized trial of technology-assisted goal setting to improve physical activity among primary care patients with prediabetes. Prev Med Rep; 2016;4:107-12.
28. McKenzie AL, Athinarayanan SJ, McCue JJ, Adams RN, Keyes M, McCarter JP, et al. Type 2 Diabetes Prevention Focused on Normalization of Glycemia: A Two-Year Pilot Study. Nutrients; 2021;13(3).

29. Morales Febles R, Marrero Miranda D, Jiménez Sosa A, González Rinne A, Cruz Perera C, Rodríguez-Rodríguez AE, et al. Exercise and Prediabetes After Renal Transplantation (EXPRED-I): A Prospective Study. Sports Med Open; 2023;9(1):32.
30. Moravcová K, Karbanová M, Bretschneider MP, Sovová M, Ožana J, Sovová E. Comparing Digital Therapeutic Intervention with an Intensive Obesity Management Program: Randomized Controlled Trial. Nutrients; 2022;14(10).
31. Muralidharan S, Ranjani H, Anjana RM, Gupta Y, Ambekar S, Koppikar V, et al. Change in cardiometabolic risk factors among Asian Indian adults recruited in a mHealth-based diabetes prevention trial. Digit Health; 2021;7:20552076211039032.
32. Nanditha A, Thomson H, Susairaj P, Srivanichakorn W, Oliver N, Godsland IF, et al. A pragmatic and scalable strategy using mobile technology to promote sustained lifestyle changes to prevent type 2 diabetes in India and the UK: a randomised controlled trial. Diabetologia; 2020;63(3):486-96.
33. Nicklas JM, Zera CA, England LJ, Rosner BA, Horton E, Levkoff SE, et al. A web-based lifestyle intervention for women with recent gestational diabetes mellitus: a randomized controlled trial. Obstet Gynecol; 2014;124(3):563-70.
34. Patel MS, Polsky D, Small DS, Park SH, Evans CN, Harrington T, et al. Predicting changes in glycemic control among adults with prediabetes from activity patterns collected by wearable devices. NPJ Digit Med; 2021;4(1):172.
35. Peacock AS, Bogossian FE, Wilkinson SA, Gibbons KS, Kim C, McIntyre HD. A Randomised Controlled Trial to Delay or Prevent Type 2 Diabetes after Gestational Diabetes: Walking for Exercise and Nutrition to Prevent Diabetes for You. Int J Endocrinol; 2015;2015:423717.
36. Petroni ML, Brodosi L, Armandi A, Marchignoli F, Bugianesi E, Marchesini G. Lifestyle Intervention in NAFLD: Long-Term Diabetes Incidence in Subjects Treated by Web- and Group-Based Programs. Nutrients; 2023;15(3).
37. Pires M, Shaha S, King C, Morrison J, Nahar T, Ahmed N, et al. Equity impact of participatory learning and action community mobilisation and mHealth interventions to prevent and control type 2 diabetes and intermediate hyperglycaemia in rural Bangladesh: analysis of a cluster randomised controlled trial. J Epidemiol Community Health; 2022;76(6):586-94.
38. Potzel AL, Gar C, Banning F, Sacco V, Fritsche A, Fritsche L, et al. A novel smartphone app to change risk behaviors of women after gestational diabetes: A randomized controlled trial. PLoS One; 2022;17(4):e0267258.
39. Ranjani H, Nitika S, Anjana R, Ramalingam S, Mohan V, Saligram N. Impact of Noncommunicable Disease Text Messages Delivered Via an App in Preventing and Managing Lifestyle Diseases: Results of the "myArogya" Worksite-Based Effectiveness Study From India. Journal of Diabetology; 2020;11(2):90-+.
40. Rollo ME, Baldwin JN, Hutchesson M, Aguiar EJ, Wynne K, Young A, et al. The Feasibility and Preliminary Efficacy of an eHealth Lifestyle Program in Women with Recent Gestational Diabetes Mellitus: A Pilot Study. Int J Environ Res Public Health; 2020;17(19).
41. Ross JAD, Barron E, McGough B, Valabhji J, Daff K, Irwin J, et al. Uptake and impact of the English National Health Service digital diabetes prevention programme: observational study. BMJ Open Diabetes Res Care; 2022;10(3).
42. Sakane N, Kotani K, Takahashi K, Sano Y, Tsuzaki K, Okazaki K, et al. Effects of telephone-delivered lifestyle support on the development of diabetes in participants at high risk of type 2 diabetes: J-DOIT1, a pragmatic cluster randomised trial. BMJ Open; 2015;5(8):e007316.
43. Salmon MK, Gordon NF, Constantinou D, Reid KS, Wright BS, Kridl TL, et al. Comparative Effectiveness of Lifestyle Intervention on Fasting Plasma Glucose in Normal Weight Versus Overweight and Obese Adults With Prediabetes. Am J Lifestyle Med; 2022;16(3):334-41.
44. Savas LA, Grady K, Cotterill S, Summers L, Boaden R, Gibson JM. Prioritising prevention: Implementation of IGT Care Call, a telephone based service for people at risk of developing type 2 diabetes. Primary Care Diabetes; 2015;9(1):3-8.

45. Sepah SC, Jiang L, Peters AL. Long-term outcomes of a Web-based diabetes prevention program: 2-year results of a single-arm longitudinal study. J Med Internet Res; 2015;17(4):e92.
46. Sevilla-Gonzalez MDR, Bourguet-Ramirez B, Lazaro-Carrera LS, Martagon-Rosado AJ, Gomez-Velasco DV, Viveros-Ruiz TL. Evaluation of a Web Platform to Record Lifestyle Habits in Subjects at Risk of Developing Type 2 Diabetes in a Middle-Income Population: Prospective Interventional Study. JMIR Diabetes; 2022;7(1):e25105.
47. Staite E, Bayley A, Al-Ozairi E, Stewart K, Hopkins D, Rundle J, et al. A Wearable Technology Delivering a Web-Based Diabetes Prevention Program to People at High Risk of Type 2 Diabetes: Randomized Controlled Trial. JMIR Mhealth Uhealth; 2020;8(7):e15448.
48. Summers C, Tobin S, Unwin D. Evaluation of the Low Carb Program Digital Intervention for the Self-Management of Type 2 Diabetes and Prediabetes in an NHS England General Practice: Single-Arm Prospective Study. JMIR Diabetes; 2021;6(3):e25751.
49. Tokunaga-Nakawatase Y, Nishigaki M, Taru C, Miyawaki I, Nishida J, Kosaka S, et al. Computer-supported indirect-form lifestyle-modification support program using Lifestyle Intervention Support Software for Diabetes Prevention (LISS-DP) for people with a family history of type 2 diabetes in a medical checkup setting: A randomized controlled trial. Prim Care Diabetes; 2014;8(3):207-14.
50. Toro-Ramos T, Michaelides A, Anton M, Karim Z, Kang-Oh L, Argyrou C, et al. Mobile Delivery of the Diabetes Prevention Program in People With Prediabetes: Randomized Controlled Trial. JMIR Mhealth Uhealth; 2020;8(7):e17842.
51. Vahlberg BM, Lundström E, Eriksson S, Holmback U, Cederholm T. Potential effects on cardiometabolic risk factors and body composition by short message service (SMS)-guided training after recent minor stroke or transient ischaemic attack: Post hoc analyses of the STROKEWALK randomised controlled trial. BMJ Open; 2021;11(10).
52. Vaughan EM, Cardenas VJ, Chan W, Amspoker AB, Johnston CA, Virani SS, et al. Implementation and Evaluation of a mHealth-Based Community Health Worker Feedback Loop for Hispanics with and at Risk for Diabetes. Journal of General Internal Medicine; 2024;39(2):229-38.
53. Wilson MG, Castro Sweet CM, Edge MD, Madero EN, McGuire M, Pilsmaker M, et al. Evaluation of a Digital Behavioral Counseling Program for Reducing Risk Factors for Chronic Disease in a Workforce. J Occup Environ Med; 2017;59(8):e150-e5.
